# The transcription factors Hsf1 and Msn2 of thermotolerant *Kluyveromyces marxianus* promote cell growth and ethanol fermentation of *Saccharomyces cerevisiae* at high temperatures

**DOI:** 10.1186/s13068-017-0984-9

**Published:** 2017-12-04

**Authors:** Pengsong Li, Xiaofen Fu, Lei Zhang, Zhiyu Zhang, Jihong Li, Shizhong Li

**Affiliations:** Institute of New Energy Technology, Tsinghua University, Beijing, 100084 China

**Keywords:** Transcription factors, *Saccharomyces cerevisiae*, *Kluyveromyces marxianus*, Ethanol fermentation, High temperatures, RNA-seq

## Abstract

**Background:**

High temperature inhibits cell growth and ethanol fermentation of *Saccharomyces cerevisiae*. As a complex phenotype, thermotolerance usually involves synergistic actions of many genes, thereby being difficult to engineer. The overexpression of either endogenous or exogenous stress-related transcription factor genes in yeasts was found to be able to improve relevant stress tolerance of the hosts.

**Results:**

To increase ethanol yield of high-temperature fermentation, we constructed a series of strains of *S. cerevisiae* by expressing 8 transcription factor genes from *S. cerevisiae* and 7 transcription factor genes from thermotolerant *K. marxianus* in *S. cerevisiae*. The results of growth curve measurements and spotting test show that *Km*Hsf1 and *Km*Msn2 can enhance cell growth of *S. cerevisiae* at 40–42 °C. According to the results of batch fermentation at 43 °C with an initial glucose concentration of 104.8 g/l, the fermentation broths of *KmHSF1* and *KmMSN2*-expressing strains could reach final ethanol concentrations of 27.2 ± 1.4 and 27.6 ± 1.2 g/l, respectively, while the control strain just produced 18.9 ± 0.3 g/l ethanol. Transcriptomic analysis found that the expression of *KmHSF1* and *KmMSN2* resulted in 55 (including 31 up-regulated and 24 down-regulated) and 50 (including 32 up-regulated and 18 down-regulated) genes with different expression levels, respectively (padj < 0.05). The results of transcriptomic analysis also reveal that *Km*Hsf1 might increase ethanol production by regulating genes related to transporter activity to limit excessive ATP consumption and promote the uptake of glucose; while *Km*Msn2 might promote ethanol fermentation by regulating genes associated with glucose metabolic process and glycolysis/gluconeogenesis. In addition, *Km*Msn2 might also help to cope with high temperature by regulating genes associated with lipid metabolism to change the membrane fluidity.

**Conclusions:**

The transcription factors *Km*Hsf1 and *Km*Msn2 of thermotolerant *K. marxianus* can promote both cell growth and ethanol fermentation of *S. cerevisiae* at high temperatures. Different mechanisms of *Km*Hsf1 and *Km*Msn2 in promoting high-temperature ethanol fermentation of *S. cerevisiae* were revealed by transcriptomic analysis.

**Electronic supplementary material:**

The online version of this article (10.1186/s13068-017-0984-9) contains supplementary material, which is available to authorized users.

## Background

The existing ethanol production has been the foundation of the transition of some countries away from a fossil fuel economy, especially for Brazil and the United States. Sugarcane ethanol has been recognized as the crowning biofuel economy around the world so far, due to its high-energy balance and significant reduction of greenhouse gas (GHG) emissions [1]. However, the sugarcane ethanol model is not suitable for every region or country of the world because of the requirements of rigorous agronomic conditions for sugarcane cultivation. Moreover, conventional ethanol production from sugarcane is based upon liquid fermentation, which requires an energy-intensive process for juice extraction, consequently resulting in tremendous energy input and the issue of wastewater disposal. Meanwhile, sweet sorghum, a sugarcane-like and fast-growing energy crop, has been considered as the most promising feedstock for biofuel production due to its much wider adaptability to climate zones, stronger tolerance to adversity, and much higher biomass yield compared to sugarcane [2]. Nevertheless, the sponge-like pith of sweet sorghum obviously lowers its crushing rate, milling performance, and cost efficiency for production of sweet sorghum ethanol compared to that of sugarcane [3–5].

To address these challenges, we have developed a novel advanced solid-state fermentation (ASSF) technology to produce ethanol using sweet sorghum stems, which is equipped with optimized and redesigned rotary drum fermenter and a proprietary yeast strain [6–9]. Low efficiencies of mass and heat transfer are the fundamental constraints of solid-state fermentation (SSF) for its industrial application [9, 10]. The ASSF technology largely improves the mass and heat transfer efficiencies during sweet sorghum ethanol production, first demonstrating that SSF can be applied at industrial scale for ethanol production [7]. Although improved rotary drum fermenter can increase the ethanol productivity from sweet sorghum to a great extent, there is still substantial reaction heat from metabolic activities of microorganisms trapped within the solid matrix due to the low thermal conductivities of chopped sweet sorghum, which always makes the temperature of SSF system to be over 40 °C, especially for the ASSF-driven ethanol plants that resided in tropical zones. Industrial ethanol fermentation typically employed mesophilic microorganisms whose optimal growth temperatures range from 25 to 37 °C. High temperature can destroy cytoskeletal integrity, because cell morphological abnormalities, inhibit cell division and growth, and impact metabolic activity [11]. Usually a cooling system is necessary for protecting microorganisms from heat stress [12], which increases complexity and cost of a fermentation system, particularly the SSF system. Thus, improving thermotolerance and fermentation efficiency of yeast cells at high temperatures would provide a cost-effective means for ethanol production.

Complex phenotypes such as thermotolerance usually involve synergistic actions of many genes and are difficult to engineer [13, 14]. Previous studies used mutagenesis or adaption evolution to improve the thermotolerance of microorganisms. However, those methods are time-consuming because the appearance of mutations is infrequent and most of the mutations are detrimental or neutral [15]. Since the exhibition of microbial complex phenotypes is largely dependent on transcription factors (TFs) that control the flow of genetic information from DNA to mRNA, TFs can be candidates to be engineered to improve complex phenotypes so that gene networks and cellular metabolism can be reprogramed [16, 17]. Global transcription machinery engineering (gTME) has become a promising strategy to evolve complex phenotypes in recent years [17–19]. Though effective, this method is still time-consuming and labor-intensive because construction of a random mutant library and high-throughput screening are needed throughout this technology.

Recent studies have proven that the overexpression of either endogenous or exogenous stress-related TFs in yeast cells can improve stress tolerance of host cells [16, 20]. Thermotolerant microorganisms that can live under high temperature provide great insights for the development of robust strains [11]. In response to heat stress, thermotolerant microorganisms can activate signaling pathways that lead to expressions of heat shock or heat stress proteins such as molecular chaperones, ubiquitin, etc. [21, 22]. The thermotolerant yeast *Kluyveromyces marxianus* has been attracting increasing attention due to its extraordinary thermotolerance, high growth rate and broad substrate spectrum [23–25]. However, *K. marxianus* has a poor ethanol tolerance compared to *S. cerevisiae* [26, 27], which restricts its application for ethanol production at high temperatures. Several stress-related TFs of *S. cerevisiae* which are significantly perturbed by thermal stress at high temperature have been identified recently [28]. In the present study, we constructed a series of strains of *S. cerevisiae* by expressing stress-related TFs from *S. cerevisiae* and thermotolerant *K. marxianus.* The results of cell growth profiling and batch fermentation at high temperatures indicate that transcription factors *Km*Hsf1 and *Km*Msn2 from *K. marxianus* promoted both cell growth and ethanol fermentation of *S. cerevisiae*. Different regulatory mechanisms of *Km*Hsf1 and *Km*Msn2 were revealed by transcriptomic analysis based on RNA-seq. This study shed light on a new potential strategy for improvement of microbial complex phenotypes such as tolerance to stress or inhibitors.

## Results and discussion

### TSH3’s performance at high temperatures

*Saccharomyces cerevisiae* TSH3’s performances of growth and ethanol fermentation were assessed at different temperatures using BY4743 as a reference strain. Additional file 1: Figure S1 show the growth curves of these two strains at 30, 37, 40 and 42 °C. At all these temperatures, TSH3 grew faster than BY4743 did, its final OD_600_ values in stationary phases were higher than those of BY4743. Batch fermentation experiments were also conducted to assess the ethanol production capacity of these two strains. The fermentation data are listed in Additional file 1: Table S1. TSH3 exhibited higher ethanol fermentation capacity than BY4743 at all the temperatures tested. Especially at 42 °C, after 24 h of fermentation with an initial glucose concentration of 118.5 g/l, the fermentation broth of TSH3 reached a final ethanol concentration of 36.6 ± 0.8, which was 58.4% higher than that of BY4743. All the above results indicate that TSH3 have significant advantages in both cell growth and ethanol fermentation at high temperatures compared with BY4743. Therefore, TSH3 was used as the host strain in the following parts of this study.

### Construction of TSH3-based strains expressing stress-related TFs

To determine stress-related TFs in *K. marxianus*, we did protein–protein BLAST with the accession numbers of stress-related TFs in *S. cerevisiae* as inputs. For each stress-related TF in *S. cerevisiae* in this study, a homologue from *K. marxianus* was found except Msn4. The information of all the stress-related TFs in this study is listed in Table 1. Then a series of plasmids containing TFs-P2A-GFP co-expression cassettes were constructed and transformed into TSH3, respectively (Additional file 1: Figure S2). The P2A peptide between a target TF and the GFP functions as a *cis*-acting hydrolase element, mediating “cleavage” between the two proteins, which makes them to be generated separately from one open reading frame [29]. This method can minimize the influence of GFP because a TF can exercise its function as a single protein instead of a GFP-fused one. The successful expression of a target TF gene could be confirmed by observing the fluorescence of GFP. The results of fluorescence microscopy showed that the green fluorescence was observed from all the strains expressing TFs-P2A-GFP (Additional file 2), indicating that these strains were successfully constructed.

**Table 1:** Information of stress-related TFs

TFs	Size	Origin	Accession numbers
*Sc*Hsf1	833 aa	*S. cerevisiae* TSH3	NP_011442.3*
*Sc*Msn2	704 aa	*S. cerevisiae* TSH3	NP_013751.1*
*Sc*Msn4	630 aa	*S. cerevisiae* TSH3	NP_012861.1*
*Sc*Sfp1	683 aa	*S. cerevisiae* TSH3	DAA09702.1*
*Sc*Rpn4	531 aa	*S. cerevisiae* TSH3	DAA11830.1*
*Sc*Gcn4	281 aa	*S. cerevisiae* TSH3	NP_010907.3*
*Sc*Cst6	587 aa	*S. cerevisiae* TSH3	DAA08512.1*
*Sc*Snf2	1703 aa	*S. cerevisiae* TSH3	NP_014933.3*
*Km*Hsf1	666 aa	*K. marxianus* DMKU3-1042	BAO38270.1
*Km*Msn2	709 aa	*K. marxianus* DMKU3-1042	BAO40339.1
*Km*Sfp1	700 aa	*K. marxianus* DMKU3-1042	BAO40481.1
*Km*Rpn4	618 aa	*K. marxianus* DMKU3-1042	BAO37805.1
*Km*Gcn4	367 aa	*K. marxianus* DMKU3-1042	BAO41052.1
*Km*Cst6	513 aa	*K. marxianus* DMKU3-1042	BAO38982.1
*Km*Snf2	1646 aa	*K. marxianus* DMKU3-1042	BAO40949.1

*Sc, Saccharomyces cerevisiae*; *Km, Kluyveromyces marxianus*

* Accession numbers were from *S. cerevisiae* S288c because TSH3 has not been resequenced yet

### Cell growth profiling

**Fig. 1:**
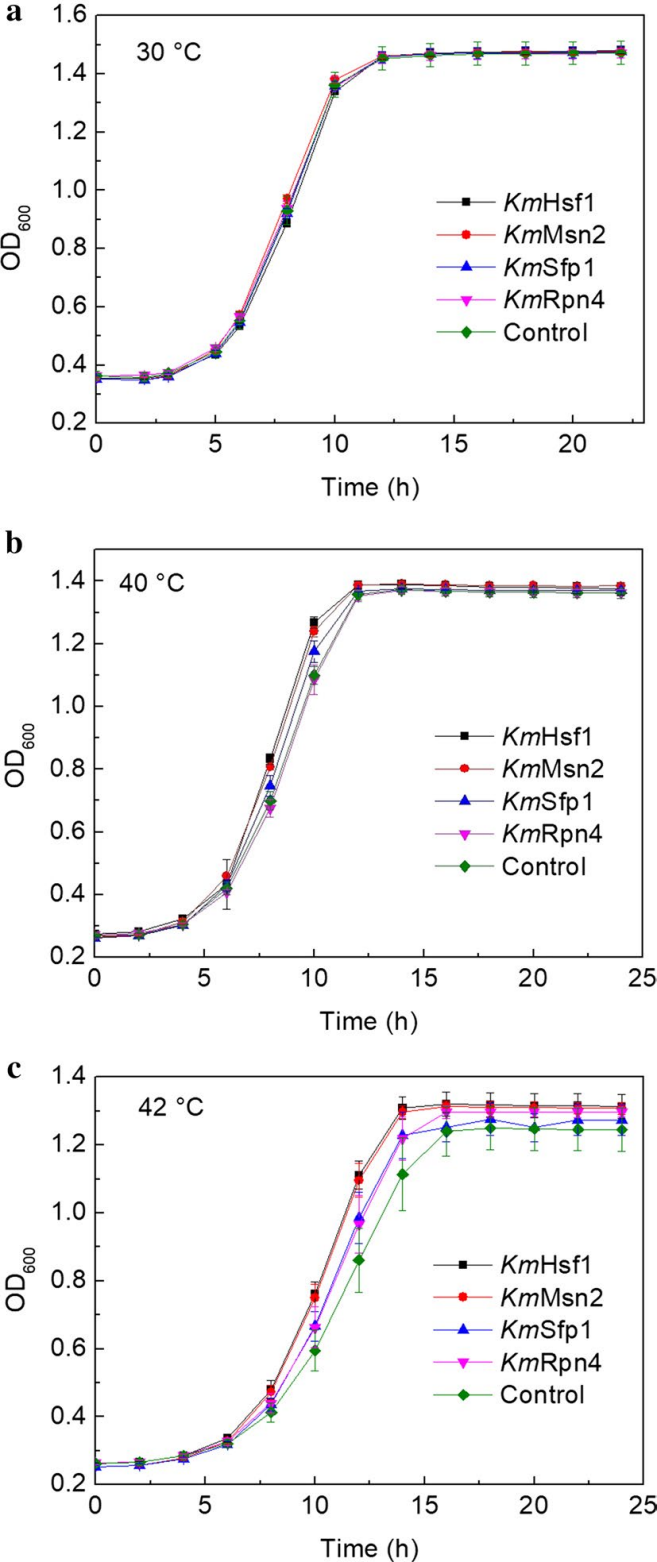
Growth curves of *S. cerevisiae* cells expressing *KmHSF1*, *KmMSN2*, *KmSFP1* and *KmRPN4* at **a** 30 °C, **b** 40 °C and **c** 42 °C. Overnight cultures of *S. cerevisiae* grown at 30 °C were diluted with YPD medium to reach an initial OD_600_ of 0.20. These cell suspensions were aliquoted in quadruplicate into a sterile 96-well plate with 200 μl in each well and incubated at 30, 40 or 42 °C in a microplate reader to measure the growth curves

To determine the key TFs that can enhance cell growth of *S. cerevisiae* at high temperatures, we first measured the growth curves of all the strains that expressing different TF genes. The growth curves of different strains at 30, 40 and 42 °C are shown in Fig. 1. There was no significant difference in the growth of all the strains at 30 °C (Fig. 1a and Additional file 1: Figure S3). At both 40 and 42 °C, however, the strains, respectively, expressing *KmHSF1* and *KmMSN2* grew the fastest, followed by the strains expressing *KmSFP1* and *KmRPN4* (Fig. 1b, c and Additional file 1: Figures S4, S5). Especially at 42 °C, both *KmHSF1*- and *KmMSN2*-expressing strains reached stationary phase at around 12 h after inoculation, earlier than other strains by about 4 h (Fig. 1c and Additional file 1: Figure S5). We also performed a spotting test to further verify the results of growth curves. All the strains were cultured to exponential growth phase and diluted with YPD medium to reach an initial OD_600_ of 0.20 and serially diluted cells were spotted onto a YPD medium plate. Similar to the growth curve results, the spots of all the strains showed no obvious difference between each other at 30 °C, while the strains expressing *KmHSF1*, *KmMSN2*, *KmSFP1* and *KmRPN4* were more resistant to high temperature than other strains at both 40 and 42 °C (Additional file 1: Figure S6). Both the growth curve and spotting test results indicate that expression of *KmHSF1*, *KmMSN2*, *KmSFP1* and *KmRPN4* conferred more thermotolerance on *S. cerevisiae*. Caspeta and Nielsen [30] found that thermotolerant yeast strains adapted by laboratory evolution showed growth trade-off at ancestral temperatures below 34 °C. In the present study, however, no trade-off in growth was found at 30 °C, indicating that the strategy used in this study was superior to adaption engineering to some extent.

**Fig. 2:**
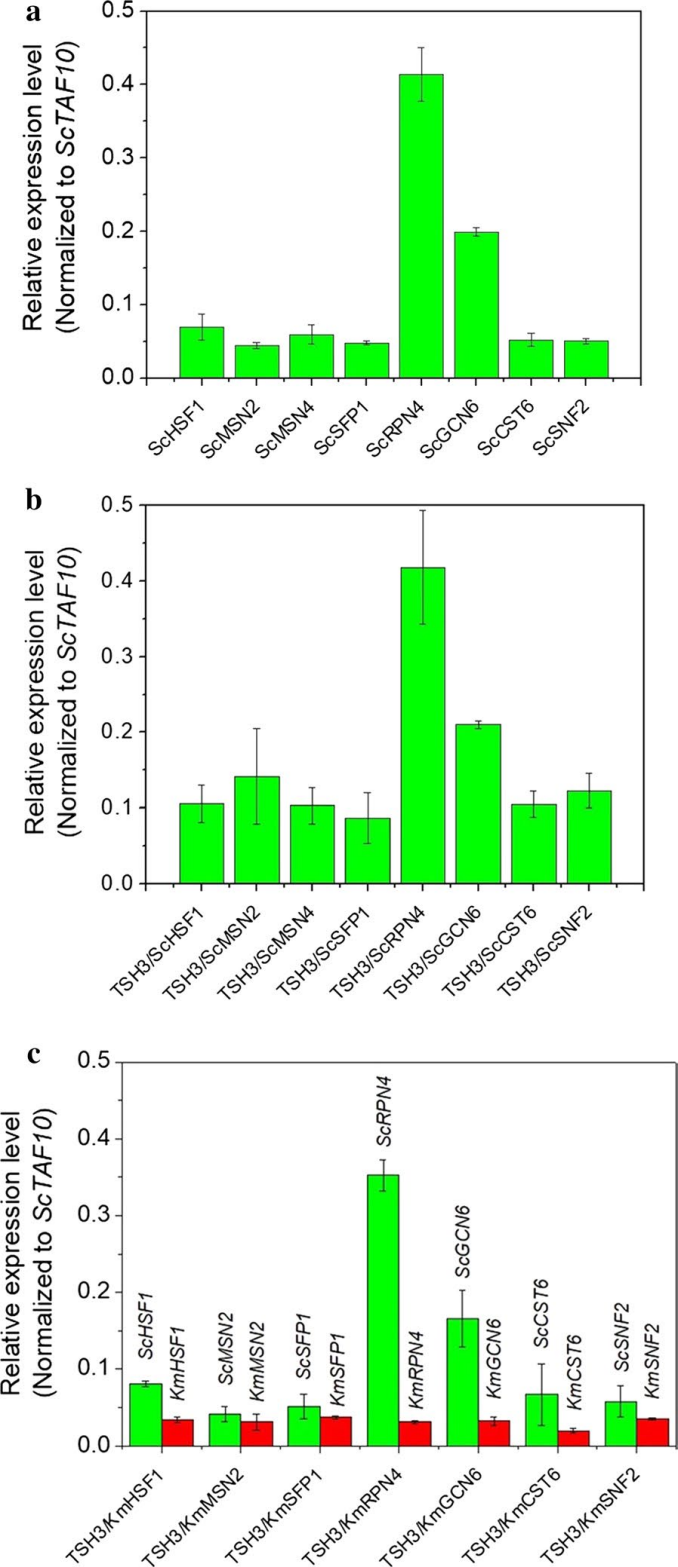
Results of qRT-PCR. **a** Relative expression levels (normalized to *ScTAF10*) of *Sc*TF genes in the control strain. **b** Relative expression levels (normalized to *ScTAF10*) of *Sc*TF genes in corresponding strains. **c** Relative expression levels (normalized to *ScTAF10*) of *Sc*TF and *Km*TF genes in corresponding strains. The strains cultured at 42 °C were used for total RNA extraction. *Sc*TF, native TF from TSH3; *Km*TF, TF from *K. marxianus* DMKU3-1042; TSH3/*TF,* the strain expressing corresponding TF gene

To assess the expression levels of the TF genes during high-temperature growth, qRT-PCR (real-time quantitative reverse transcription PCR) experiments were performed using the strains cultured at 42 °C for total RNA extraction. The gene *ScTAF10*, which encodes the Taf10 subunit of the TFIID complex, was selected as the reference gene due to its stable expression under different conditions [31]. Figure 2a shows the relative expression levels (normalized to *ScTAF10*) of the native TF genes in the control strain. The expression levels of these native TF genes were slightly increased after overexpression (Fig. 2b). Figure 2c demonstrates the relative expression levels (normalized to *ScTAF10*) of *Sc*TF and *Km*TF genes in corresponding strains. These results indicate that the expression levels of the TF genes carried in plasmids were relatively low compared with those of the native TF genes in the genome of the control strain. As genes encoding trans-acting elements, however, the TF genes from *K. marxianus* (*KmHSF1*, *KmMSN2*, *KmSFP1* and *KmRPN4*) with such low-level expression were sufficient to promote cell growth at high temperatures (Fig. 1b, c and Additional file 1: Figures S4, S5).

### Batch ethanol fermentation

Based on the results of growth curve measurements and spotting test, we performed batch fermentation experiments at 30, 40, 42 and 43 °C using the strains expressing *KmHSF1*, *KmMSN2*, *KmSFP1* and *KmRPN4*, with the strain expressing only *GFP* gene as a control. The fermentation data are listed in Table 2. The strains expressing *KmHSF1* and *KmMSN2* showed significantly improved ethanol fermentation performance at 40, 42 and 43 °C (*P* < 0.05) (Additional file 3). Especially at 43 °C, after 24 h of fermentation with an initial glucose concentration of 104.8 g/l, the fermentation broths of *KmHSF1*- and *KmMSN2*-expressing strains reached final ethanol concentrations of 27.2 ± 1.4 and 27.6 ± 1.2 g/l, which were 43.6 and 45.6% higher than the control group, respectively. Based on the theoretical maximum yield of 0.51 g ethanol/g glucose, the metabolic yield of *KmHSF1*- and *KmMSN2*-expressing strains were 0.38 g ethanol/g glucose (74.9% of the theoretical yield) and 0.38 g ethanol/g glucose (74.7% of the theoretical yield), respectively. The results of the fermentation experiments indicate that the transcription factors *Km*Hsf1 and *Km*Msn2 of *K. marxianus* promote ethanol fermentation of *S. cerevisiae* at high temperatures.

**Table 2:** Fermentation results to evaluate the ethanol production potential of strains harboring candidate stress-related TFs (sampling time: 24 h; initial concentration of glucose: 104.8 g/l)

	Control	*Km*Hsf1		*Km*Msn2		*Km*Sfp1		*Km*Rpn4	
			Percent improvement (%)		Percent improvement (%)		Percent improvement (%)		Percent improvement (%)
Final ethanol concentration (g/l)									
30 °C	41.8 ± 1.3	41.8 ± 1.4	0.2	42.0 ± 0.7	0.5	42.5 ± 0.7	1.8	42.1 ± 1.4	0.7
40 °C	35.6 ± 1.1	39.1 ± 1.3	9.6	39.2 ± 1.2	10.1	36.9 ± 1.3	3.7	36.4 ± 0.5	2.4
42 °C	25.3 ± 1.3	30.5 ± 1.2	20.4	31.0 ± 0.6	22.6	25.9 ± 1.5	2.4	25.5 ± 1.3	1.3
43 °C	18.9 ± 0.3	27.2 ± 1.4	43.6	27.6 ± 1.2	45.7	19.7 ± 1.0	4.1	16.2 ± 0.8	− 14.6
Consumed glucose (g/l)									
30 °C	103.6 ± 0.9	103.7 ± 1.0	0.1	104.7 ± 0.1	1.1	104.7 ± 0.1	1.1	103.7 ± 0.4	0.2
40 °C	88.5 ± 0.7	96.7 ± 0.8	9.3	97.1 ± 0.5	9.7	90.5 ± 0.4	2.2	90.3 ± 0.7	2.0
42 °C	60.9 ± 1.6	73.3 ± 2.0	20.2	74.1 ± 1.8	21.6	63.4 ± 1.2	3.8	61.9 ± 1.6	1.6
43 °C	52.2 ± 1.4	71.1 ± 0.8	36.3	72.3 ± 0.8	38.6	52.3 ± 0.7	0.2	48.0 ± 0.9	− 8.0
Metabolic yield (g ethanol/g glucose)									
30 °C	0.40	0.40	0.03	0.40	− 0.6	0.41	0.7	0.41	0.5
40 °C	0.40	0.40	0.3	0.40	0.4	0.40	0.5	0.41	2.2
42 °C	0.42	0.42	0.2	0.42	0.8	0.41	− 1.3	0.41	− 0.9
43 °C	0.36	0.38	5.3	0.38	5.1	0.38	3.9	0.34	− 7.2
Percentage of the theoretical yield: 0.51 g ethanol/g glucose (%)									
30 °C	79.1%	79.1%	0.03	78.6%	− 0.6	79.6%	0.7	79.5%	0.5
40 °C	78.8%	79.0%	0.3	79.1%	0.4	79.9%	1.4	79.1%	0.4
42 °C	81.4%	81.5%	0.2	82.1%	0.8	80.3%	− 1.3	80.7%	− 0.9
43 °C	71.1%	74.9%	5.3	74.7%	5.1	73.9%	3.9	66.0%	− 7.2

Both Hsf1 and Msn2 in eukaryotes are responsible for heat stress induced gene expression [32, 33]. Hsf1 is engaged in both constitutive and stress-inducible DNA binding, regulating expression of genes involved in protein folding and degradation, and other broad range of biological functions [34, 35]. Msn2 is a general stress transcription factor, the overexpression of which in *S. cerevisiae* could also confer resistance to various stresses such as furfural [36] and ethanol stress [37]. Both Hsf1 and Msn2 can bind to specific *cis*-acting elements in the promoter regions using their DNA-binding domains (DBDs) to activate the expression of corresponding genes [38, 39]. The *cis*-acting elements that Hsf1 and Msn2 bind to are called heat shock element (HSE) and stress response promoter element (STRE), respectively [40]. After binding to these *cis*-acting elements, the transactivation domains of Hsf1 and Msn2 can activate the expression of corresponding genes. A recent study has proven that even single amino acid changes in a DBD can switch its DNA-binding specificity [41]. Therefore, the DBDs of Hsf1 and Msn2 are crucial for their DNA-binding specificity. The sequence alignment results show that there are some gaps and amino acid mutations between *Sc*Hsf1 and *Km*Hsf1 as well as between *Sc*Msn2 and *Km*Msn2 (Additional file 1: Figures S7, S8). Therefore, we can speculate that *Km*Hsf1 and *Km*Msn2 could probably induce the expression of some key genes of *S. cerevisiae* that the native TFs cannot.

### Transcriptomic analysis

To reveal the regulatory mechanisms of *Km*Hsf1 and *Km*Msn2 on ethanol fermentation of *S. cerevisiae* at high temperatures, the transcriptional profiles of strains expressing *KmHSF1* (KH_43) and *KmMSN2* (KM_43) were investigated using transcriptomic analysis based on RNA-seq with three biological replicates.

**Fig. 3:**
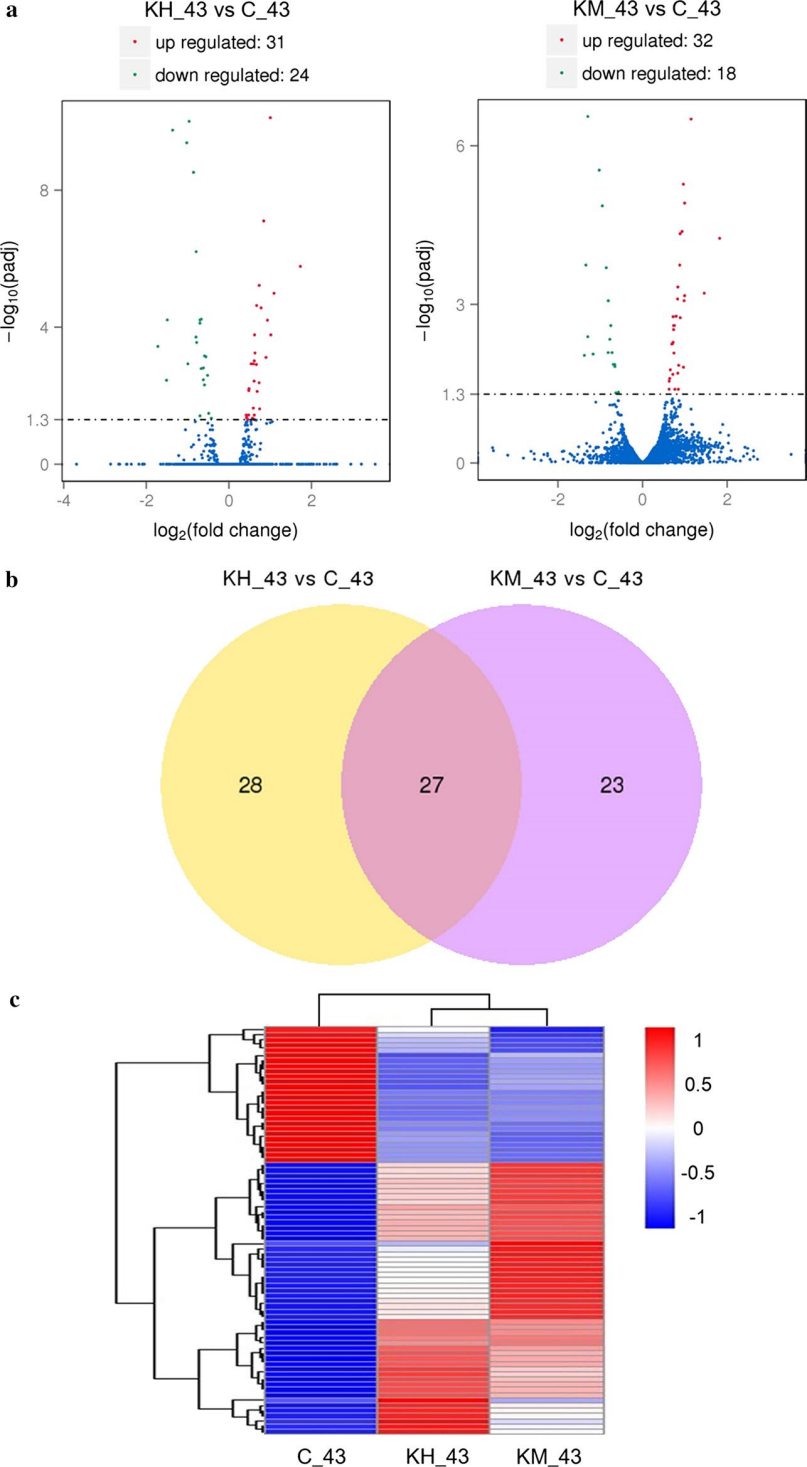
Differential expression analysis. **a** Volcano plots of DEGs in KH_43 vs C_43 and KM_43 vs C_43. **b** Venn diagram showing the overlap of differentially expressed genes. **c** Clustering analysis of differentially expressed genes. KH_43: Batch fermentation of the stain expressing *KmHSF1* at 43 °C; KM_43: batch fermentation of the strain expressing *KmMSN2* at 43 °C; C_43: batch fermentation of the control strain at 43 °C

According to differential expression analysis of RNA-seq data, KH_43 was identified to have 55 differentially expressed genes (DEGs) (including 31up-regulated and 24 down-regulated) compared to the control C_43 (Fig. 3a), while KM_43 had 50 DEGs (including 32 up-regulated and 18 down-regulated) (Fig. 3a). Among all these DEGs, 27 genes were found differentially expressed in both KH_43 and KM_43 (Fig. 3b). Supporting information on the detailed description of DEGs is provided in Additional file 4. Furthermore, the DEGs were selected for clustering analysis, which helps to understand the relationships and discrepancy of samples more intuitively and comprehensively. The same types of genes were gathered in a cluster with similar biological functions. Compared with gene expression pattern of the control, those of KH_43 and KM_43 were more similar to each other (Fig. 3c), indicating that *Km*Hsf1 and *Km*Msn2 have similar functions in gene transcriptional regulation to some extent.

**Fig. 4:**
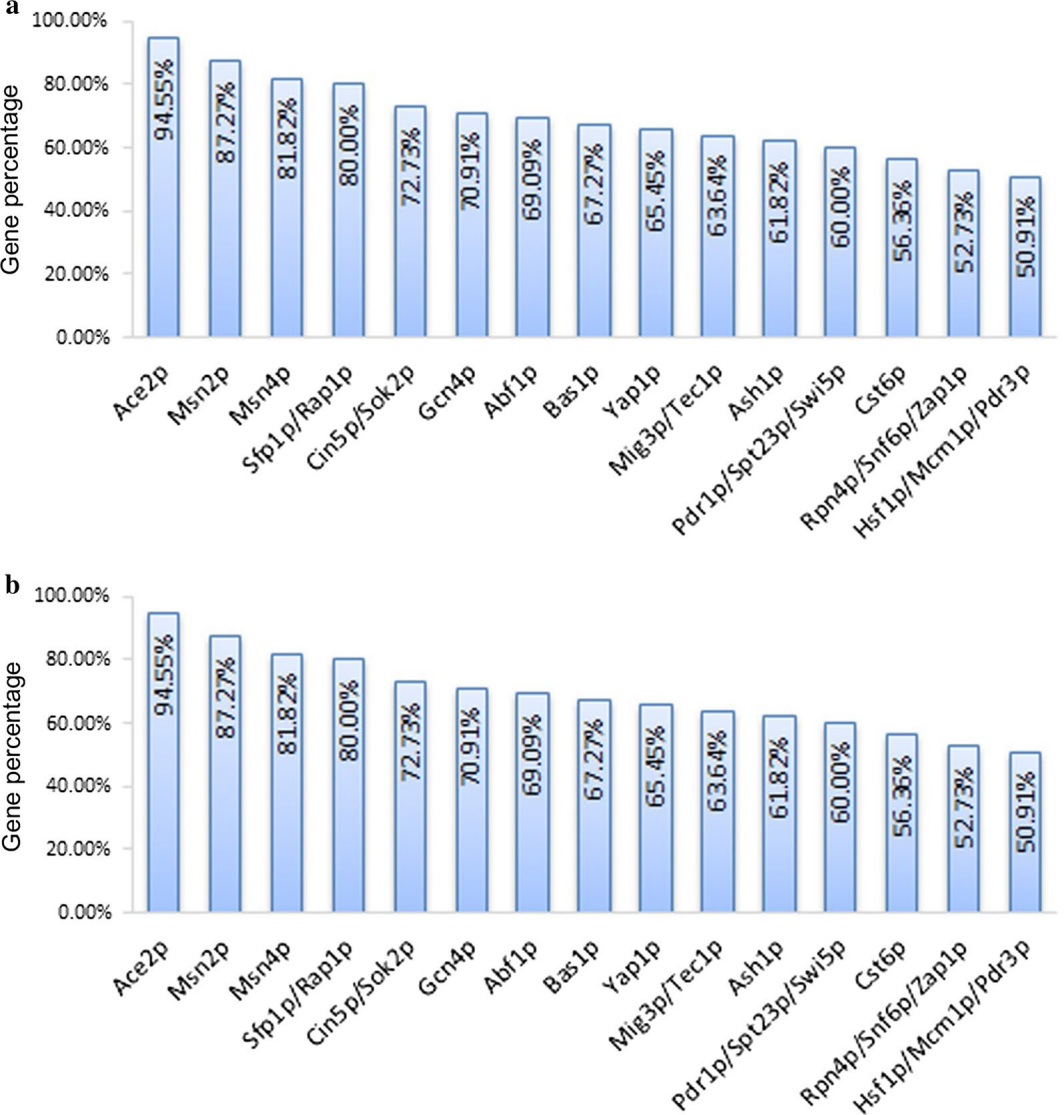
Transcription factor (TF) profiles for regulating the DEGs in **a** KH_43 and **b** KM_43. The percentage of genes regulated by each of the top 20 TFs was calculated as the number of genes regulated by the TF relative to the total number of DEGs involved in response to *Km*Hsf1 or *Km*Msn2. KH_43: Batch fermentation of the stain expressing *KmHSF1* at 43 °C; KM_43: batch fermentation of the strain expressing *KmMSN2* at 43 °C

Transcription factor analysis was conducted to identify the transcription factors that are most likely involved in regulating yeast transcriptome and to find the transcription factors that have the most similar gene regulatory pattern to that of *Km*Hsfp1 and *Km*Msn2. TF profiles were generated by choosing the top 15 candidates based on the coverage of genes they regulated (Fig. 4). For both KH_43 and KM_43, Ace2 and Msn2 were the top two TF candidates, indicating that both *Km*Hsfp1 and *Km*Msn2 have similar regulating pattern to Ace2 and Msn2 of *S. cerevisiae*. In *S. cerevisiae*, Ace2 participates in regulating the life cycle and carbon metabolism of the cells [42], while Msn2 is involved in stress response [16]. According to the result of sequence alignment, the sequences of *Sc*Ace2, *Sc*Msn2, *Km*Hsf1 and *Km*Msn2 do show some homology to each other (Additional file 1: Figure S9), which implies they can probably bind similar *cis*-acting elements in the promoters to activate gene expression. The TFs *Sc*Ace2 and *Sc*Msn2 were also identified to be the top TFs that involved in regulating consensus genes related to stress response to acetic and furfural in *S. cerevisiae* by Chen et al. [16], indicating that *S. cerevisiae* may has similar regulatory mechanisms in response to those chemicals and heat stress. However, a complex phenotype involves synergistic actions of many genes and two different TFs can hardly activate the same set of genes by binding the *cis*-acting elements in their promoters, that is why *Sc*Msn2 did not lead to thermotolerance as *Km*Hsf1 and *Km*Msn2 did.

To identify the function of DEGs, we conducted Gene Ontology (GO) and KEGG enrichment analysis. Based on GO enrichment analysis, transporter activity (GO:0005215) was enriched in DEGs for KH_43 vs C_43 (corrected *P* < 0.05) (Additional file 5: Table S2). Six genes encoding various transporters were up-regulated. For example, Hsp30 is a stress-inducible regulator of plasma membrane H^+^-ATPase that can provide an energy conservation role, thus the up-regulated expression of *HSP30* could limit excessive ATP consumption by plasma membrane H^+^-ATPase during prolonged heat stress exposure [43]. *FCY2* encodes a purine-cytosine permease which mediates purine (adenine, guanine, and hypoxanthine) and cytosine accumulation. *FCY2* was also found up-regulated under heat stress in another study [44]. *HXT6* that encodes a high-affinity glucose transporter was up-regulated, while the genes encoding low-affinity and moderate-affinity glucose transporters, *HXT1* and *HXT5*, were down-regulated, which could probably promote ethanol fermentation by increasing the uptake of glucose.

For KM_43 vs C_43, monocarboxylic acid metabolic process (GO:0032787), glucose metabolic process (GO:0006006) and monocarboxylic acid biosynthetic process (GO:0072330) were enriched (corrected *P* < 0.05) (Additional file 5: Table S3). As to glucose metabolic process (GO:0006006), genes encoding enzymes required for ethanol production, such as *ENO1*, *PGI1*, *ADH1*, *TDH1 and TDH3*, were up-regulated, while *ALD6* which encodes a cytosolic aldehyde dehydrogenase Ald6 was down-regulated. Since Ald6 is required for conversion of acetaldehyde to acetate, which is a side reaction of ethanol fermentation [45, 46], its down-regulated expression could benefit ethanol production. Regarding monocarboxylic acid metabolic process (GO:0032787) and monocarboxylic acid biosynthetic process (GO:0072330), genes associated with the biosynthesis of long-chain saturated fatty acids, monounsaturated fatty acids and sterol, were up-regulated. For instance, *FAS1* encodes the beta subunit of fatty acid synthetase that catalyzes the synthesis of long-chain saturated fatty acids [47]; *OLE1* encodes the delta (9) fatty acid desaturase that is required for monounsaturated fatty acid synthesis [48]. Yeast can adapt different temperature by changing fatty acid composition, degree of unsaturation and the mean fatty acid chain length [48, 49]. In addition, *ERG3*, encoding C-5 sterol desaturase that catalyzes the introduction of a C-5 double bond into episterol [50], was also up-regulated. The up-regulation of *ERG3* could substantially change the sterol composition in the membrane. Recent studies have found that the change in sterol metabolism could help to improve the thermotolerance of yeast, indicating that regulation of type and amount of sterols has a significant modulatory role and serves as an adaptive response to temperature variations [30, 51–53]. Both fatty acids and sterols are main constituents of cell membrane lipids and their changes can affect membrane fluidity, thereby rendering yeast more thermotolerant. Thus, *Km*Msn2 could probably help to cope with high temperature by regulating genes associated with lipid metabolism to change the membrane fluidity.

According to KEGG enrichment analysis, glycolysis/gluconeogenesis (KEGG pathway sce01100) was enriched in DEGs for KM_43 vs C_43 (corrected *P* < 0.05) (Additional file 5: Table S4). However, KEGG enrichment analysis failed to enrich pathways for KH_43 vs C_43 when choosing a corrected *P* value less than 0.05, which was probably due to the small number of DEGs. Glycolysis is a process of glucose oxidation in which each glucose molecule can be broken down into two pyruvate molecules. Then the pyruvate formed by glycolysis is converted into ethanol and CO_2_ in two steps catalyzed by pyruvate decarboxylase and alcohol dehydrogenase, respectively, i.e., ethanol fermentation [54]. Therefore, it is can be deduced that *Km*Msn2 could promote the glycolysis process at high temperatures to some extent, thereby enhancing ethanol fermentation.

**Fig. 5:**
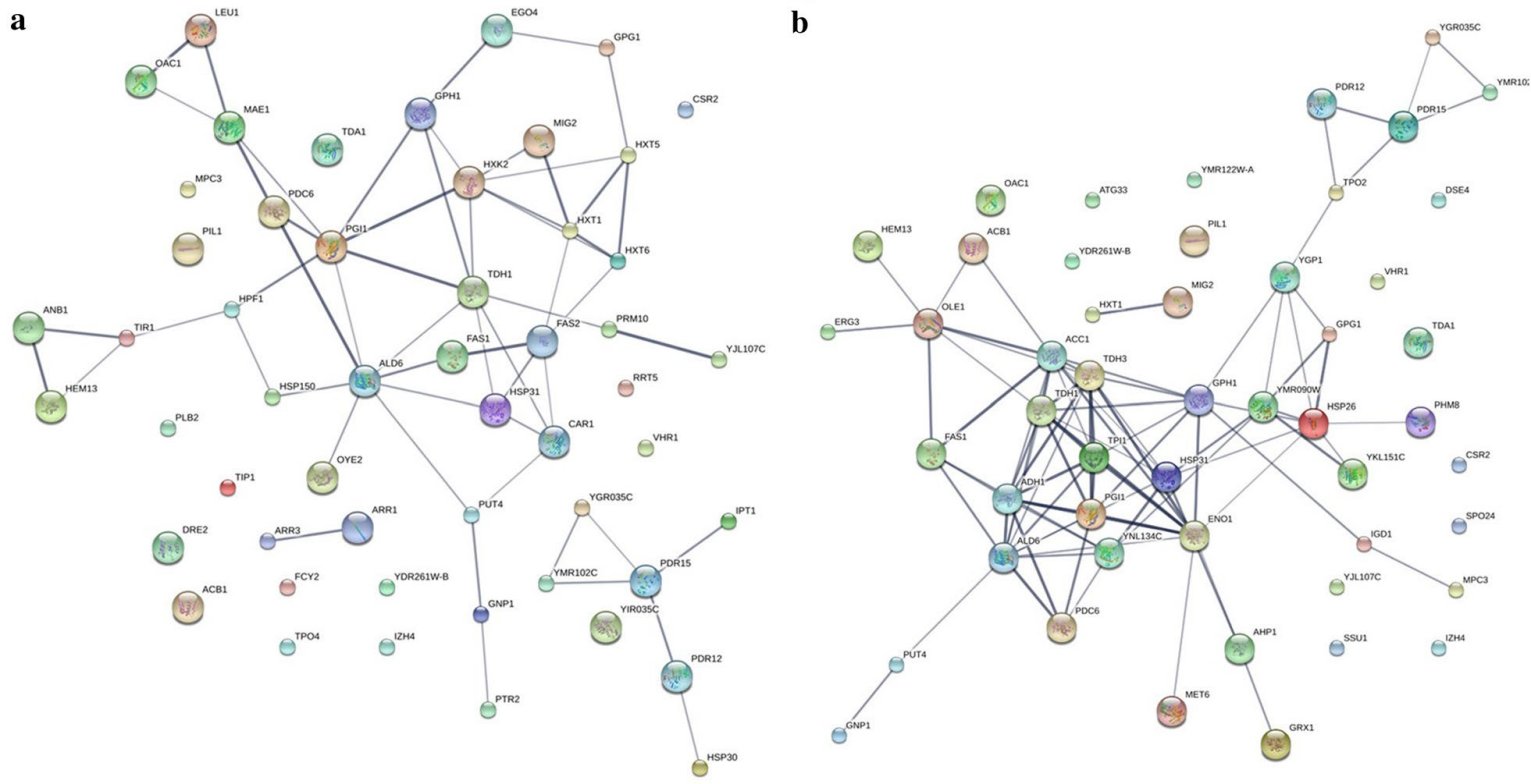
Interaction network of DEGs in **a** KH_43 and **b** KM_43. (score > 0.5) Interactions are indicated by edges, with thicker edges having stronger associations. KH_43: Batch fermentation of the stain expressing *KmHSF1* at 43 °C; KM_43: batch fermentation of the strain expressing *KmMSN2* at 43 °C

The interaction of the identified DEGs are integrated and predicted in the STRING database (http://string-db.org/) [55]. Figure 5 shows the different interaction networks of the DEGs in KH_43 and KM_43. In the DEGs in KH_43, genes related to glycolysis/gluconeogenesis (such as *ALD6*, *HXK2*, *PDC6*, *PGI1*, *TDH1*) and glucose transport (such as *HXK2*, *HXT1*, *HXT5, HXT6*) have close interactions (confidence score > 0.5), respectively (Fig. 5a). Regarding the DEGs in KM_43, genes associated with glycolysis/gluconeogenesis (such as *ADH1*, *ALD6*, *ENO1*, *PDC6*, *PGI1*, *TDH1*, *TDH3*, *TPI1*) and lipid metabolism (such as *OLE1*, *ERG3*, *ACB1*, *ACC1*, *FAS1*) have close interactions (confidence score > 0.5), respectively (Fig. 5b). These results, together with the results of GO and KEGG analysis, reveal that *Km*Hsf1 regulate gene expressions involved in glycolysis/gluconeogenesis and glucose transport, while *Km*Msn2 regulate gene expressions related to glycolysis/gluconeogenesis and lipid metabolism.

## Methods

### Strains, plasmids and media

*Escherichia coli* TOP10 (Tiangen, Beijing, China) was used as a host for DNA cloning and plasmid propagation. *Kluyveromyces marxianus* DMKU3-1042 (purchased from NITE Biological Resource Center with the deposit number of NBRC 104275) was used for genomic DNA isolation and gene amplification. *Saccharomyces cerevisiae* TSH3, which was isolated from the stalk surface of SO_2_-treated sweet sorghum and shows high ethanol productivity at high temperatures (up to 43 °C) or in the presence of high level SO_2_ under SSF, was used for genomic DNA isolation, gene amplification, fermentation experiments and RNA extraction. The commonly used yeast strain *S. cerevisiae* BY4743 (a/α*his3Δ/his3Δ leu2Δ/leu2Δ* +*/lys2Δ met15Δ/*+ *ura3Δ/ura3*Δ) [6] was used as a reference strain to benchmark TSH3’s performance at high temperatures. The plasmid pScLP2 was constructed based on pAUR123 (Takara, Japan), with the selectable marker *AUR1*-*C* replaced by G418-resistant gene *KanMX6*. *E. coli* was grown in LB medium (1% tryptone, 0.5% yeast extract, 1% NaCl) containing 100 μg/ml ampicillin. *Saccharomyces cerevisiae* was grown in YPD medium (1% yeast extract, 2% peptone and 2% glucose), with 200 μg/ml G418 sulfate added for strains transformed with pScLP2-based vectors. Fermentation medium (FM) [20 g/l peptone, 20 g/l yeast extract, 100 g/l glucose, 0.6 g/l (NH_4_)_2_SO_4_, 0.15 g/l KH_2_PO_4_] was used for batch fermentation experiments.

### Determination of target TFs

Stress-related TFs of *S. cerevisiae* were determined based on the work by Shui et al. [28] *K. marxianus* TFs that are homologous to those of *S. cerevisiae* were determined through NCBI (National Center for Biotechnology Information) online tool protein–protein BLAST (blastp, https://blast.ncbi.nlm.nih.gov/Blast.cgi), using accession numbers of *S. cerevisiae* TFs as inputs.

### DNA manipulation

EZNA^®^ Yeast DNA Kit (Omega Bio-tek, Doraville, CA, USA) was used to isolate the genomic DNA of *S. cerevisiae* and *K. marxianus*, following the supplier’s protocol. Concentration of isolated DNA was measured with a spectrophotometer at 260 nm (Nanodrop). The *TF*-*GFP* co-expression plasmids were constructed using the method described by Szymczak-Workman, et al. [56]. A fragment containing a *Sma*I site, a Kozak sequence, a *Sc*Hsf1 gene without stop codon, the coding sequence of a GSG linker and the 5′ region of a 2A peptide derived from porcine teschovirus-1 (P2A) [57, 58] in turn was amplified with the oligonucleotides *Sc*Hsf1-F and *Sc*Hsf1-R. Another fragment containing 3′ region of P2A, a GFP gene and an *Xho*I site in turn was amplified with oligonucleotides GFP-F and GFP-R. Then the two resulting fragments were connected via a final overlap PCR with the oligonucleotides *Sc*HSF1-F and GFP-R as primers, forming the fragment *ScHSF1*-*P2A*-*GFP*. *ScHSF1*-*P2A*-*GFP* was then cloned into the shuttle plasmid pScLP2 after digested with restriction enzymes *Sma*I and *Xho*I. The genes coding for other stress-related TFs were amplified using the oligonucleotides listed in additional file 6 and cloned into the pScLP2-based vector, replacing *ScHSF1* via one-step sequence- and ligation-independent cloning (SLIC) [59]. *S. cerevisiae* TSH3 cells were transformed with the resulting plasmids containing *TF*-*P2A*-*GFP* cassettes *S.c.* EasyComp transformation kit (Thermo Fisher Scientific, USA).

### Microscopy

Yeast strains transformed with *TF*-*P2A*-*GFP* expressing plasmids were grown exponentially in liquid YPD media containing 200 mg/l G418 sulfate and were washed three times in PBS buffer (136.89 mM NaCl, 8.09 mM Na_2_HPO_4_, 1.76 mM KH_2_PO_4_, 2.68 mM KCl) before microscopy. Then the cells were viewed by both bright-field and fluorescence microscopy using an Axio Vert.A1 microscope (Carl Zeiss, Göttingen, Germany) equipped with an AxioCam HRc CCD camera (Carl Zeiss, Germany).

### Cell growth profiling

Cultures of *S. cerevisiae* strains expressing different TFs were grown in YPD medium containing 200 mg/l G418 sulfate. Overnight cultures of *S. cerevisiae* grown at 30 °C with shaking at 200 rpm were diluted with YPD medium to reach an initial OD_600_ (optical density at 600 nm) of 0.20. These cell suspensions were aliquoted in quadruplicate into sterile 96-well plates with 200 μl in each well and incubated at 30, 40 or 42 °C in a Tecan Infinite M200 Pro plate reader (Tecan Group Ltd., Männedorf, Switzerland) until stationary phase was reached. Absorbance values were automatically recorded at intervals of 2 h. Before each measurement, cell cultures were automatically shaken for 90 s to homogenize the samples. For spotting test, 2 μl cell suspensions of each strain with OD_600_ of 0.20 and serial dilutions of 10^−1^–10^−3^ were spotted onto YPD agar medium and then incubated at 30 °C for 24 h, 40 °C for 72 h and 42 °C for 96 h.

### Real-time quantitative reverse transcription PCR (qRT-PCR)

Yeast cells were grown to early exponential phase and then the total RNA was extracted using EZNA^®^ Yeast RNA Kit (Omega Bio-tek, Doraville, CA, USA). First-strand of cDNA was generated from the total RNA using FastKing RT Kit (With gDNase) (Tiangen, Beijing, China). Then the generated cDNA was used as qRT-PCR templates. The gene *TAF10*, which encodes the Taf10 subunit of the TFIID complex, was selected as the reference gene [31]. The qRT-PCR-based relative quantification of TF gene transcripts in comparison to the reference gene transcript was performed using Talent qPCR PreMix (SYBR Green) (Tiangen, Beijing, China) on a Step One Plus Real-Time PCR System (Applied Biosystems, Foster City, CA, USA). The primers for all the TF genes and the reference gene were listed in Additional file 6.

### Batch ethanol fermentation

Yeast cells were pre-cultured in YPD medium containing 200 mg/l G418 sulfate overnight, washed with sterilized water, and then inoculated into fermentation media. Batch fermentation experiments were conducted under oxygen-limited conditions in sealed 100 ml serum bottle containing 30 ml media at 30, 40, 42 or 43 °C and 100 rpm. The initial cell densities were adjusted to OD_600_ = 1.0. All fermentation experiments were set up in triplicate.

### HPLC analysis

Fermentation samples were taken at intervals of 12 h, centrifuged at 14,000*g* for 10 min, and filtered with 0.45 μm filters. Concentrations of substrate and metabolites were measured using high performance liquid chromatography (HPLC) with an Aminex HPX-87H column (Bio-Rad, Hercules, CA, USA). The metabolic yield (g ethanol/g glucose) is calculated by dividing the weight of ethanol produced (g ethanol) by the weight of glucose consumed (g glucose). The ethanol conversion efficiency (%) is the percentage of metabolic yield (g ethanol/g glucose) in the theoretical maximum ethanol yield (0.51 g ethanol/g glucose).

### Protein sequence alignment

Protein sequence alignments were conducted using the National Center for Biotechnology Information (NCBI) online alignment tool COBALT (https://www.ncbi.nlm.nih.gov/tools/cobalt/). The protein accession numbers were used as the inputs.

### Sample preparation for RNA-seq

Yeast cells were grown to early exponential phase under oxygen-limited conditions in 30 mL YPD medium in 100 ml serum bottles in biological triplicate, and were cultured at 43 °C for 18 h before cell samples were collected for RNA-seq analysis. Samples taken from each replicate incubations were collected in pre-chilled Corning tubes and were centrifuged at 4 °C for 1 min. The cell pellets were flash-frozen in liquid nitrogen and stored at − 80 °C before analysis. Total RNA was extracted using the EZNA^®^ Yeast RNA Kit (Omega Bio-tek, Doraville, CA, USA). The RNA samples were then sent to Novogene Bioinformatics Technology (Beijing, China) for quality and quantity evaluation, cDNA library preparation, and sequencing.

### RNA-seq and bioinformatics analysis

To reveal the mechanisms of the thermotolerance conferred by *Km*Hsf1 and *Km*Msn2, the transcriptional profiles of TSH3/pScLP2-*KmHSF1*-*P2A*-*GFP* (KH_43), TSH3/pScLP2-*KmMSN2*-*P2A*-*GFP* (KM_43) fermenting at 43 °C were investigated using RNA-seq with three biological replicates. Considering both *KmHSF1* and *KmMSN2* were co-expressed with *GFP* in the *TF*-*P2A*-*GFP* cassette, TSH3/pScLP2-*GFP* (C_43) that only a *GFP* gene in the cassette was selected as the control. We first compared the gene expression profiles between KH_43 and C_43, KM_43 and C_43 to find the respective differentially expressed genes (DEGs) using DESeq R package [60]. The resulting P values were adjusted using the Benjamin and Hochberg’s approach for controlling the false discovery rate. Genes with an adjusted *P* value less than 0.05 found by DESeq were assigned as differentially expressed. Then the genes showing significant difference in transcription level were selected for clustering analysis which helps to understand the relationships and discrepancy of samples more comprehensively and intuitively. The same types of genes were gathered in a cluster with similar biological functions. Transcription factor analysis was conducted using a previously published method [16, 61]. The differentially expressed genes identified by the RNA-seq analysis were searched against all of the transcription factors in the YEASTRACT database (http://www.yeastract.com). The number of genes that a transcription factor can regulate in the pool of differentially expressed genes was calculated by YEASTRACT database. Then this number is divided by the total number of DEGs to calculate the percentage of genes that a TF can regulate, which is defined as the ratio of the number of differentially expressed genes the TF can regulate to the number of total differentially expressed genes. Gene Ontology (GO) enrichment analysis of DEGs was implemented by the GOseq R package [62], in which gene length bias was corrected. GO terms with corrected *P* value less than 0.05 were considered significantly enriched by DEGs. KOBAS software was used to test the statistical enrichment of DEGs in KEGG pathways [63]. The interaction networks of DEGs were obtained using the STRING v10.5 database (http://string-db.org/) [55].

## Conclusion

The transcription factors *Km*Hsf1 and *Km*Msn2 of thermotolerant *Kluyveromyces marxianus* promoted both cell growth and ethanol fermentation of *Saccharomyces cerevisiae* at high temperatures. According to the results of 24-h batch fermentation with an initial glucose concentration of 104.8 g/l, the fermentation broths of *KmHSF1* and *KmMSN2* expressing strains could reach final ethanol concentrations of 27.2 ± 1.4 and 27.6 ± 1.2 g/l, respectively, while the control strain just produced 18.9 ± 0.3 g/l ethanol. Transcriptomic analysis reveals different regulatory mechanisms of *Km*Hsf1 and *Km*Msn2 in *S. cerevisiae*. *Km*Hsf1 may increase ethanol production at high temperatures by regulating genes related to transporter activity to limit excessive ATP consumption and promote the uptake of glucose; while *Km*Msn2 may promote ethanol fermentation at high temperatures by regulating genes associated with glucose metabolic process and glycolysis/gluconeogenesis. In addition, *Km*Msn2 may also regulate genes associated with lipid metabolism, so that the membrane fluidity can be changed to cope with high temperature. This study also demonstrates that stress-related transcription factors from thermotolerant microorganisms may provide a potential resource to increase ethanol production at high temperatures.

## Additional files

**Additional file 1: Figure S1.** Growth curves of *S. cerevisiae* TSH3 and BY4743 at 30, 37, 40 and 42 °C. **Figure S2.** The map of TF-GFP co-expression plasmid pScLP2-TF-P2A-GFP. **Figures S3–S5.** Growth curves of *S. cerevisiae* cells expressing all the TF genes at 30, 40 and 42 °C. **Figure S6.** Spotting test of *S. cerevisiae* cells expressing different TF genes at 30, 40 and 42 °C. **Figure S7.** Sequence alignment between *Sc*Hsf1 and *Km*Hsf1. **Figure S8.** Sequence alignment between *Sc*Msn2 and *Km*Msn2. **Figure S9.** Sequence alignment between *Sc*Ace2, *Sc*Msn2, *Km*Hsf1 and *Km*Msn2. **Table S1.** Fermentation results of TSH3 and BY4743.

**Additional file 2.** Fluorescence microscopy of the constructed yeast strains in this study.

**Additional file 3: Sheet 1.** Statistical analysis of the growth curve data. **Sheet 2.** Statistical analysis of the fermentation data.

**Additional file 4: Sheet 1.** Differentially expressed genes for KH_43 vs C_43. **Sheet 2.** Differentially expressed genes for KM_43 vs C_43. **Sheet 3.** Consensus genes regulated by *Km*Hsf1 and *Km*Msn2.

**Additional file 5: Table S2.** GO analysis of differentially expressed genes for KH_43 vs C_43. **Table S3.** GO analysis of differentially expressed genes for KM_43 vs C_43. **Table S4.** KEGG analysis of differentially expressed genes for KM_43 vs C_43.

**Additional file 6.** Primers used in this study.

## Abbreviations

ASSF: advanced solid-state fermentation
SSF: solid-state fermentation
TFs: transcription factors
PCR: polymerase chain reaction
GFP: green fluorescent protein
YPD: yeast extract, peptone and dextrose
RNA-seq: ribonucleic acid (RNA) sequencing
DEGs: differentially expressed genes
GO: gene ontology
KEGG: Kyoto Encyclopedia of Gene and Genomics
