# Supplementary material for: The transcription factors Hsf1 and Msn2 of thermotolerant Kluyveromyces marxianus promote cell growth and ethanol fermentation of Saccharomyces cerevisiae at high temperatures
Source: Biotechnol Biofuels. 2017 Dec 4;10:289. doi: 10.1186/s13068-017-0984-9 (PMC5713069; doi:10.1186/s13068-017-0984-9)
Supplement: Supplementary file 1 — Additional file 1: Figure S1. Growth curves of S. cerevisiae TSH3 and BY4743 at 30, 37, 40 and 42 °C. Figure S2. The map of TF-GFP co-expression plasmid pScLP2-TF-P2A-GFP. Figures S3–S5. Growth curves of S. cerevisiae cells expressing all the TF genes at 30, 40 and 42 °C. Figure S6. Spotting test of S. cerevisiae cells expressing different TF genes at 30, 40 and 42 °C. Figure S7. Sequence alignment between ScHsf1 and KmHsf1. Figure S8. Sequence alignment between ScMsn2 and KmMsn2. Figure S9. Sequence alignment between ScAce2, ScMsn2, KmHsf1 and KmMsn2. Table S1. Fermentation results of TSH3 and BY4743. [file 13068_2017_984_MOESM1_ESM.docx]

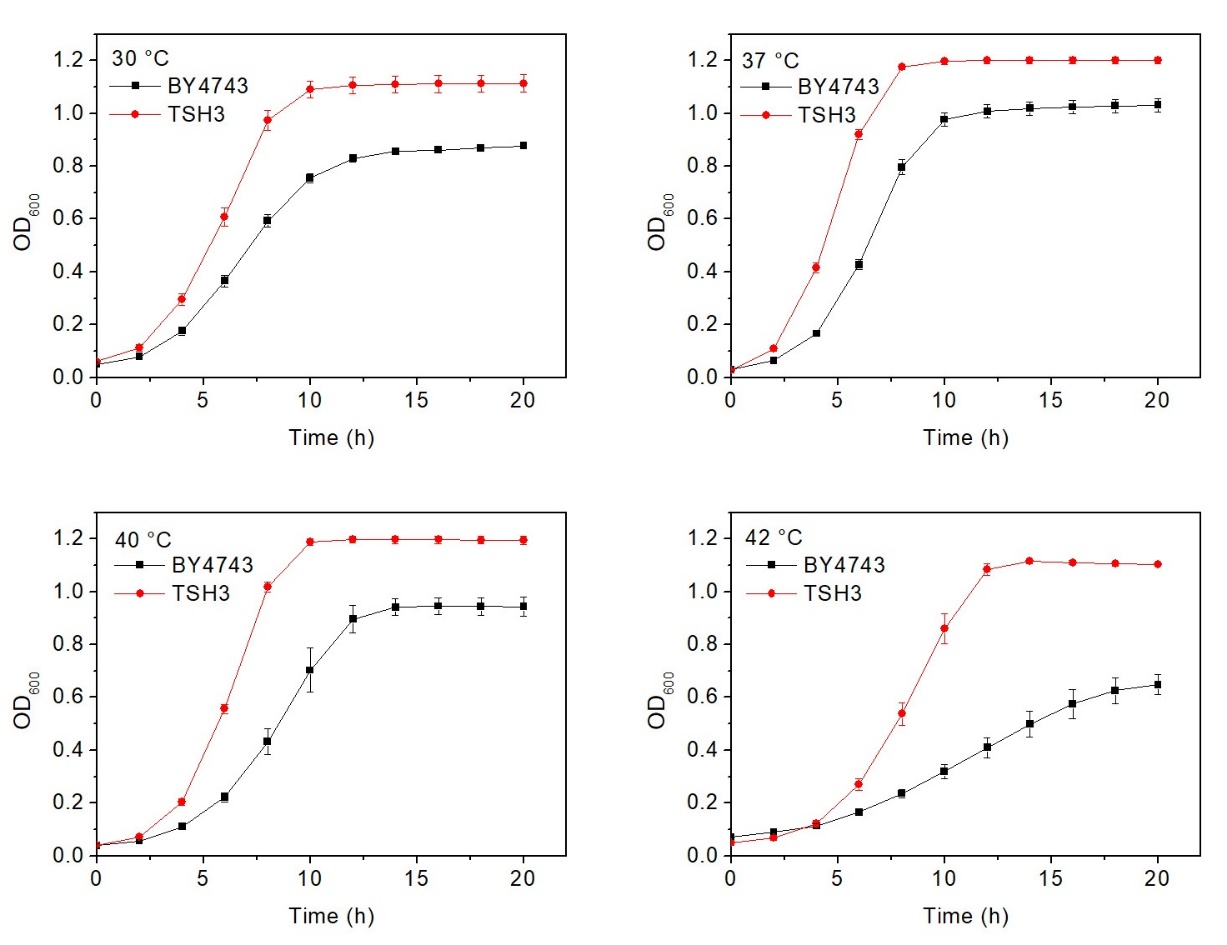


**Figure S1** Growth curves of *S. cerevisiae* TSH3 and BY4743 at 30, 37, 40 and 42 °C. Overnight cultures grown at 30 °C were diluted with YPD medium to reach an initial OD_600_ below 0.10. These cell suspensions were aliquoted in quadruplicate into a sterile 96-well plate with 200 μl in each well and incubated at 30, 37, 40 or 42 °C in a microplate reader to measure the growth curves.


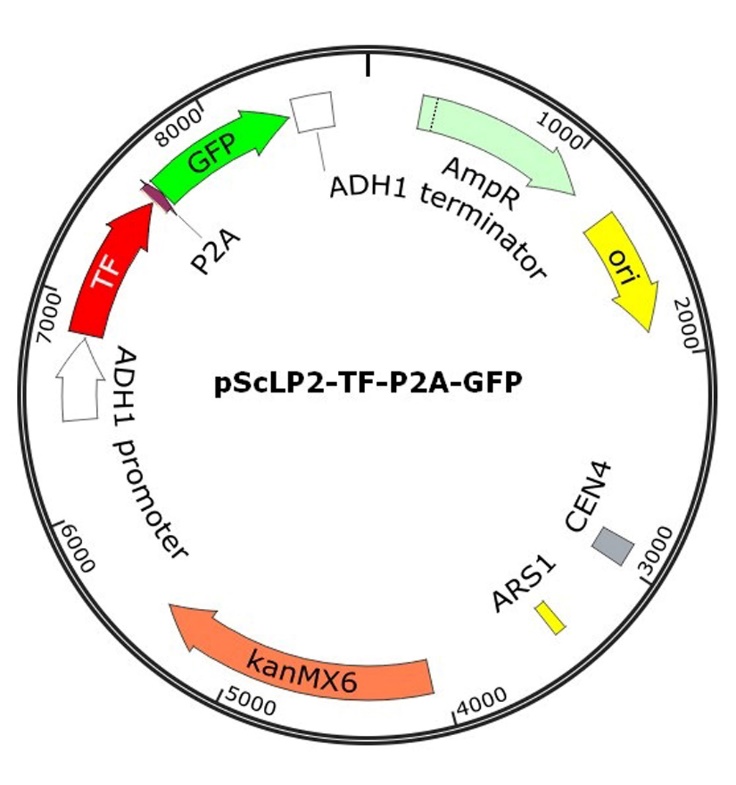


**Figure S2** The map of TF-GFP co-expression plasmid pScLP2-TF-P2A-GFP

*TF*: A transcription factor gene in this study; P2A: 2A peptide derived from porcine teschovirus-1; *GFP*: green fluorescent protein gene; *AmpR*: ampicillin-resistance gene; ori: pUC origin of replication; *CEN4*: centromere; *AR*S1: autonomous replicating sequence; *KanMX6*: G418-resistance gene.


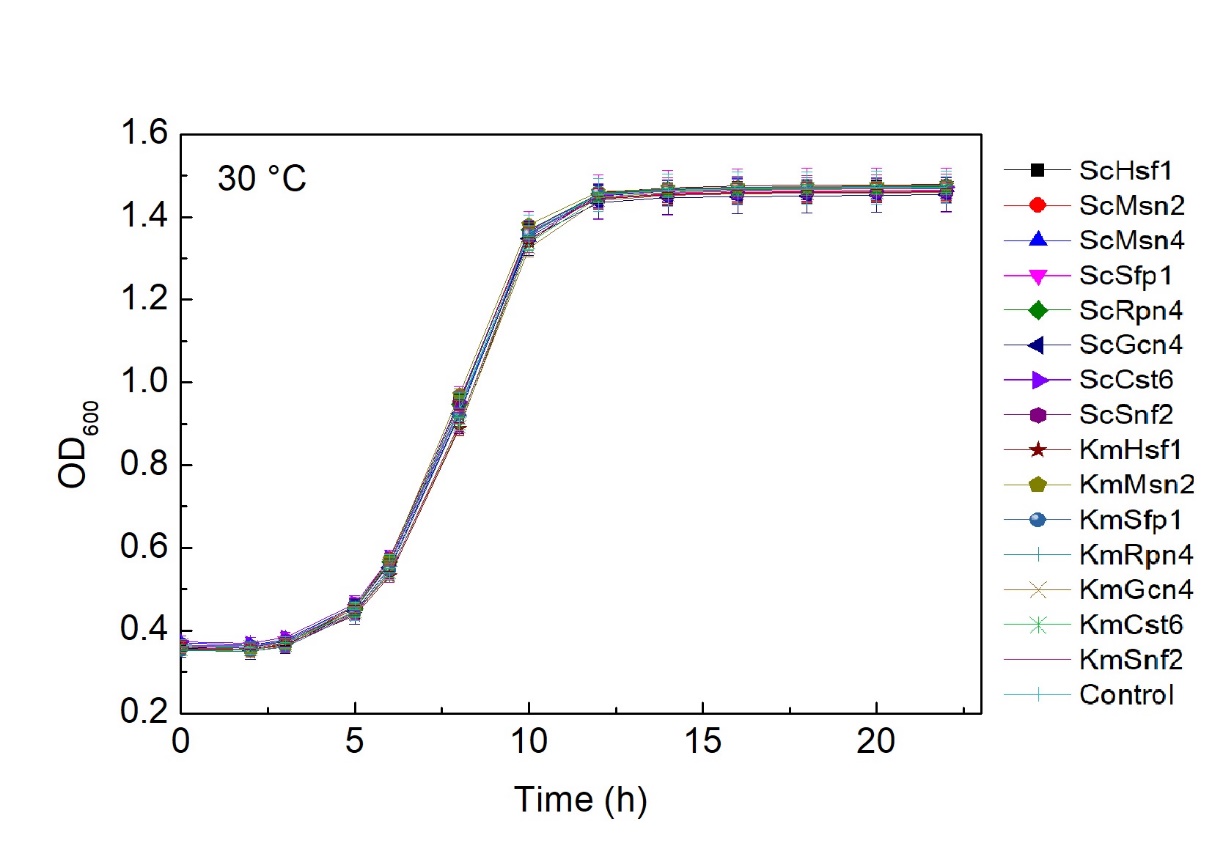


**Figure S3** Growth curves of *S. cerevisiae* cells expressing all the TF genes at 30 °C

**
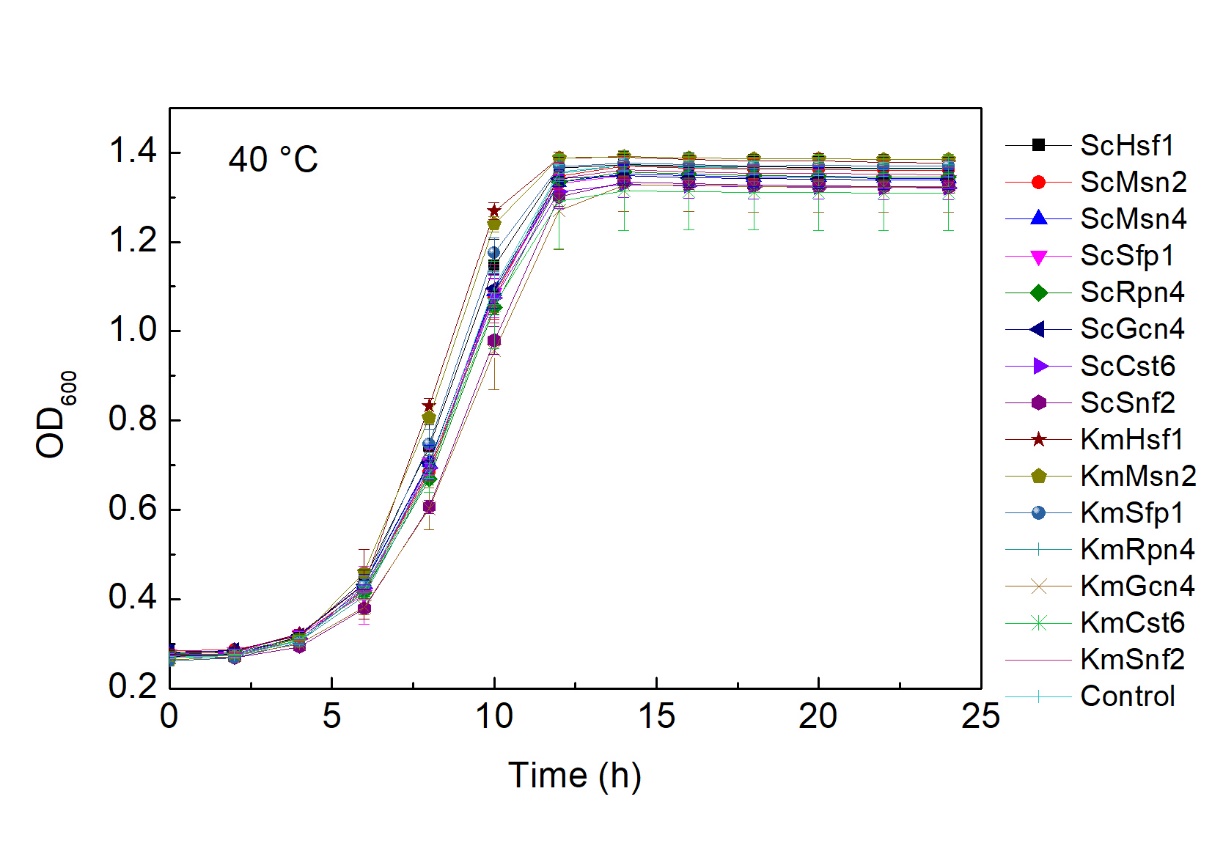
**

**Figure S4** Growth curves of *S. cerevisiae* cells expressing all the TF genes at 40 °C

**
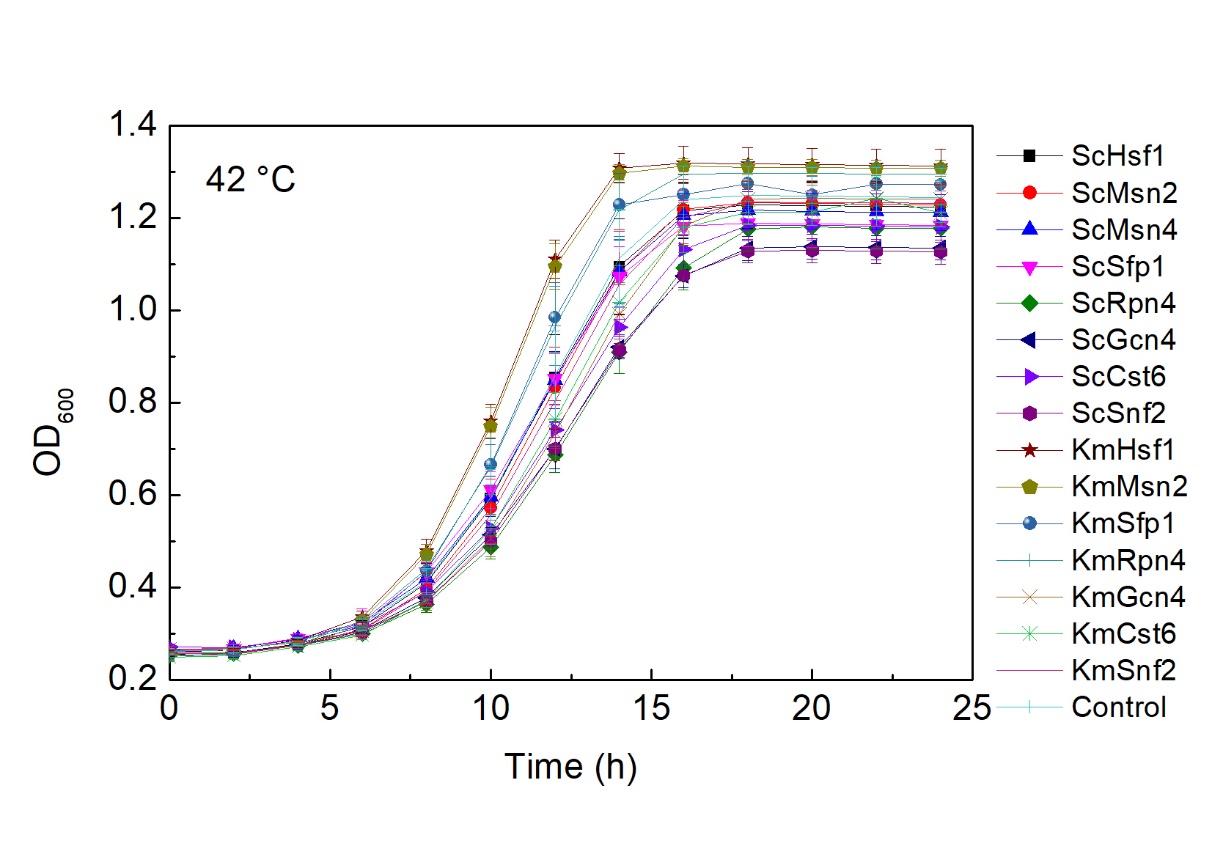
**

**Figure S5** Growth curves of *S. cerevisiae* cells expressing all the TF genes at 42 °C

**
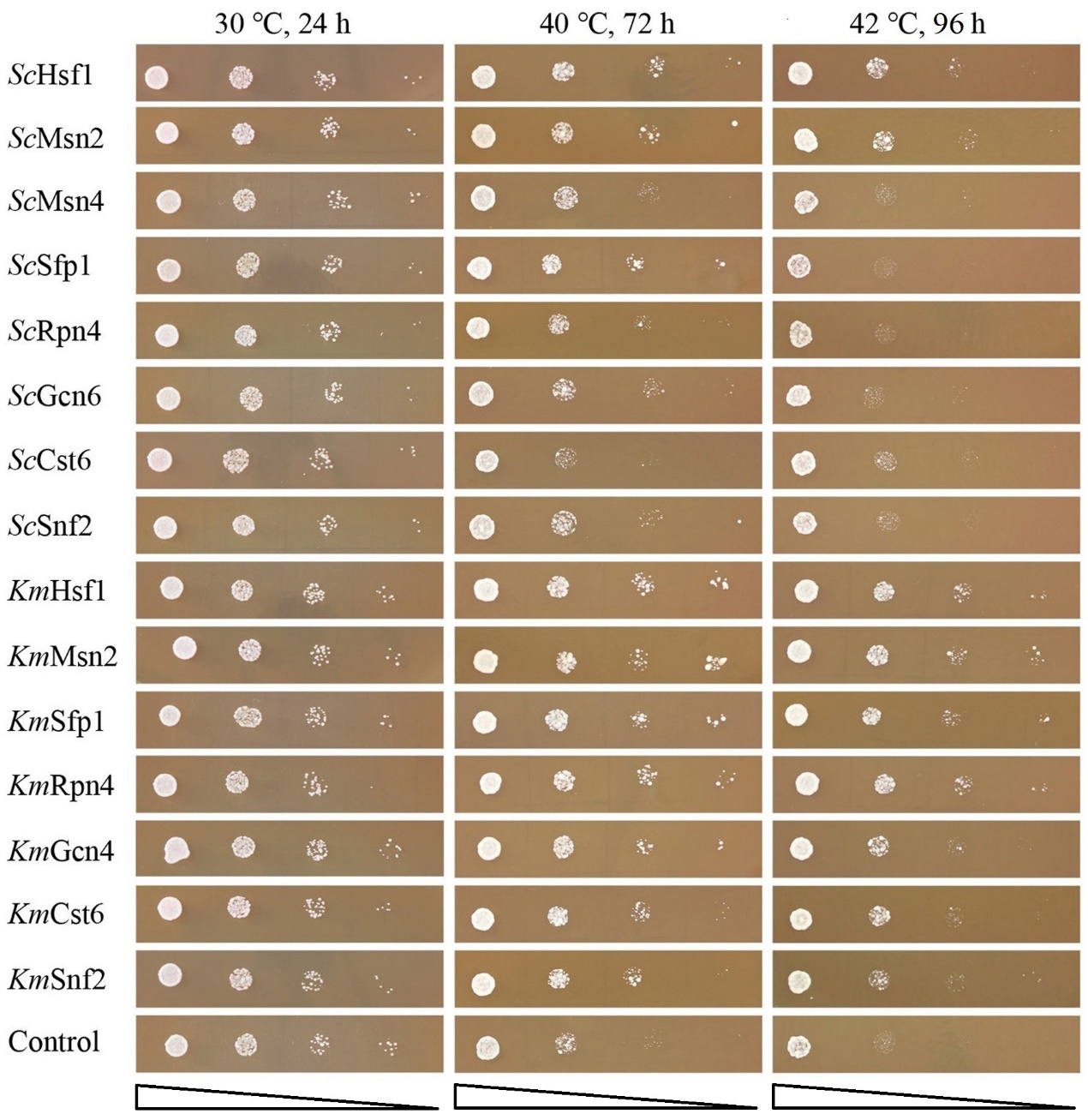
**

**Figure S6** Spotting test of *S. cerevisiae* cells expressing different TF genes at 30 °C, 40 °C and 42 °C. 2 μl cell suspensions of each strain with OD_600_ of 0.20 and serial dilutions of 10^-1^ to 10^-3^ were spotted onto YPD agar medium and then incubated at 30 °C for 24 h, 40 °C for 72 h and 42 °C for 96 h.


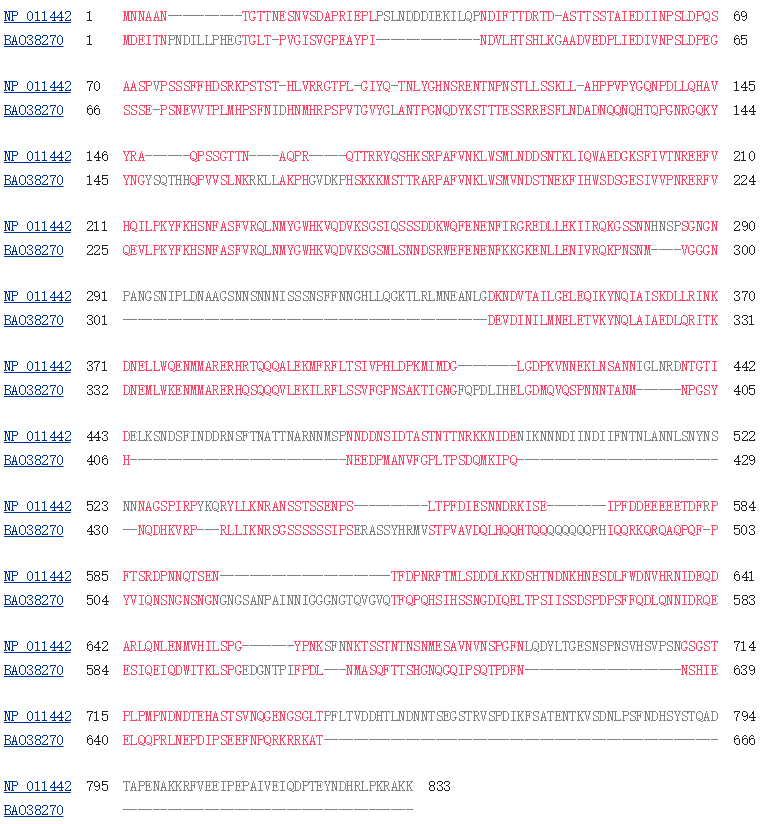


**Figure S7** Sequence alignment between *Sc*Hsf1 and *Km*Hsf1

NP_011442: *Sc*Hsf1 of *S. cerevisiae* S288c

BAO38270: *Km*Hsf1 of *K. marxianus* DMKU3-1042


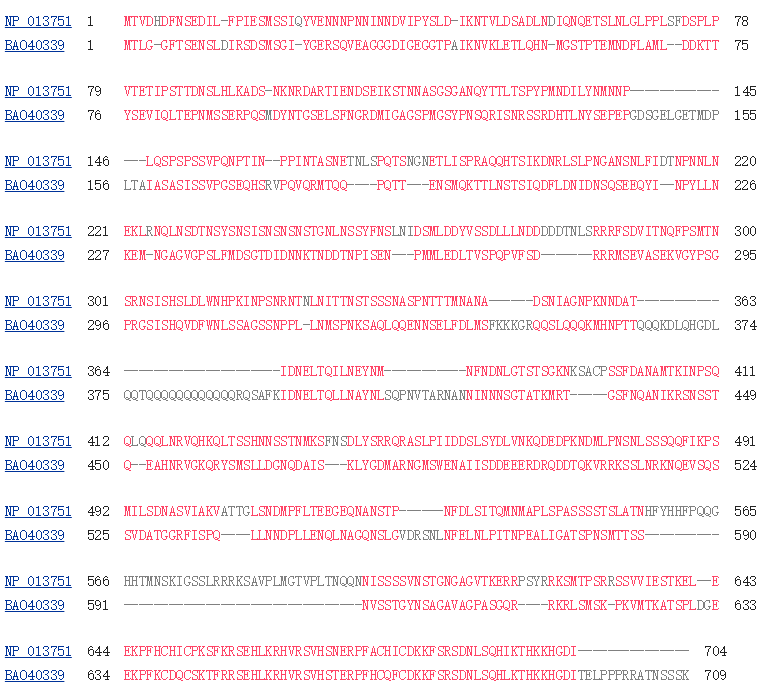


**Figure S8**  Sequence alignment between *Sc*Msn2 and *Km*Msn2

NP_013751: *Sc*Msn2 of *S. cerevisiae* S288c

BAO40339: *Km*Msn2 of *K. marxianus* DMKU3-1042

**
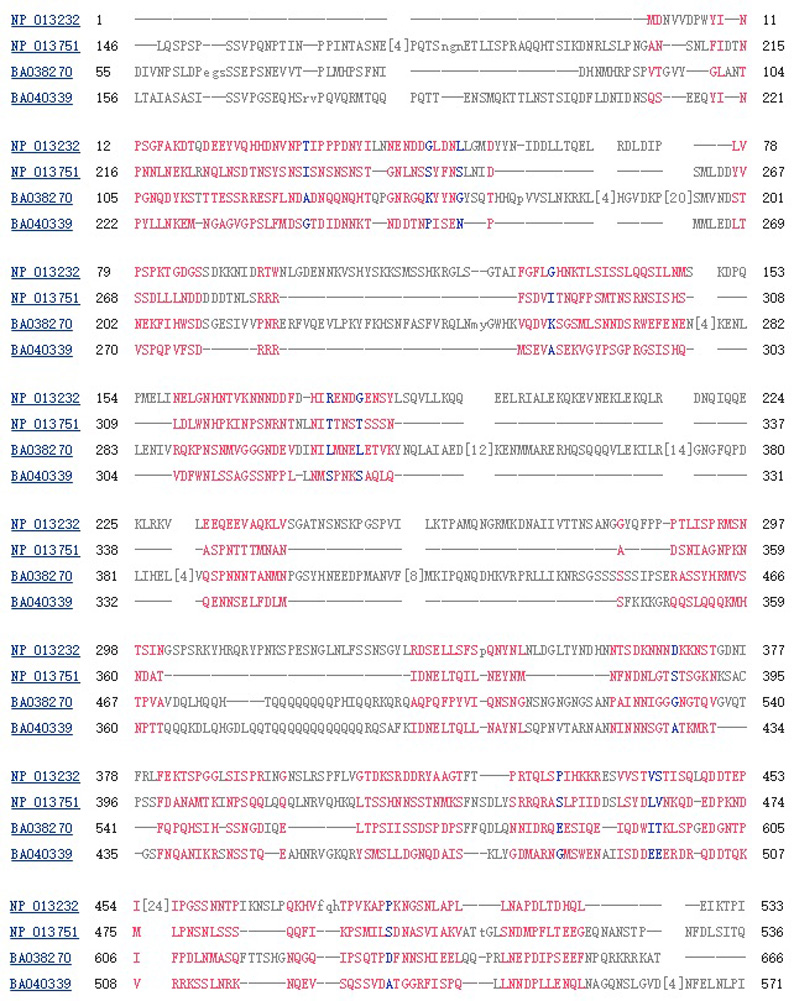
**

**Figure S9**  Sequence alignment between *Sc*Ace2, *Sc*Msn2, *Km*Hsf1 and *Km*Msn2

NP_013232: *Sc*Ace2 of *S. cerevisiae* S288c

NP_013751: *Sc*Msn2 of *S. cerevisiae* S288c

BAO38270: *Km*Hsf1 of *K. marxianus* DMKU3-1042

BAO40339: *Km*Msn2 of *K. marxianus* DMKU3-1042

**Table S1** Fermentation results of TSH3 and BY4743

(Sampling time: 24 h; initial concentration of glucose: 118.5 g/l)

|  |  | BY4743 | TSH3 | |
| --- | --- | --- | --- | --- |
|  |  |  |  | **Percent improvement** |
| **Final ethanol concentration (g/l)** | **30** **°C** | 37.2±0.5 | 52.7±0.1 | 41.7% |
|  | **37 °C** | 46.7±1.2 | 51.4±0.1 | 10.1% |
|  | **40 °C** | 38.6±0.1 | 46.5±0.3 | 20.5% |
|  | **42 °C** | 23.1±0.8 | 36.6±0.8 | 58.4% |
|  |  |  |  |  |
| **Consumed glucose**  **(g/l)** | **30 °C** | 86.8±3.7 | 118.5±0.0 | 36.5% |
|  | **37 °C** | 102.8±2.5 | 118.5±0.0 | 15.3% |
|  | **40 °C** | 88.7±0.6 | 109.6±0.6 | 23.6% |
|  | **42 °C** | 56.6±0.8 | 90.2±0.8 | 59.4% |
|  |  |  |  |  |
| **Metabolic yield (g ethanol/g glucose)** | **30 °C** | 0.43 | 0.44 | 2.3% |
|  | **37 °C** | 0.45 | 0.43 | -4.4% |
|  | **40 °C** | 0.44 | 0.42 | -4.5% |
|  | **42 °C** | 0.41 | 0.41 | 0% |
|  |  |  |  |  |
| **Percentage of the theoretical yield: 0.51 g ethanol/g glucose (%)** | **30 °C** | 84.3% | 88.3% | 2.3% |
|  | **37 °C** | 88.2% | 84.3% | -4.4% |
|  | **40 °C** | 88.3% | 82.4% | -4.5% |
|  | **42 °C** | 80.4% | 80.4% | 0% |
