# Supplementary material for: The transcription factors Hsf1 and Msn2 of thermotolerant Kluyveromyces marxianus promote cell growth and ethanol fermentation of Saccharomyces cerevisiae at high temperatures
Source: Biotechnol Biofuels. 2017 Dec 4;10:289. doi: 10.1186/s13068-017-0984-9 (PMC5713069; doi:10.1186/s13068-017-0984-9)
Supplement: Supplementary file 2 — Additional file 2. Fluorescence microscopy of the constructed yeast strains in this study. [file 13068_2017_984_MOESM2_ESM.docx]

**Fluorescence microscopy of the constructed yeast strains in this study:**

| **Expressed gene** | **Bright-field microscopy** | **Fluorescence microscopy** |
| --- | --- | --- |
| *ScHSF1-P2A-GFP* | 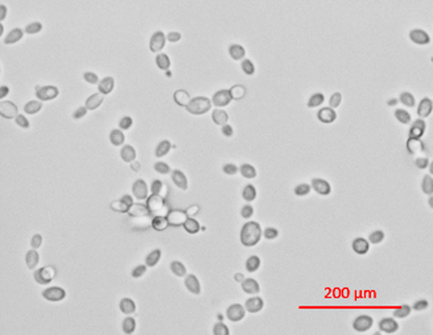 | 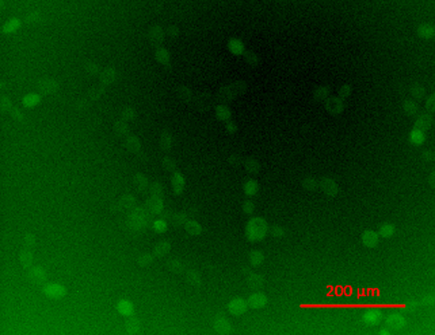 |
| *ScMSN2-P2A-GFP* | 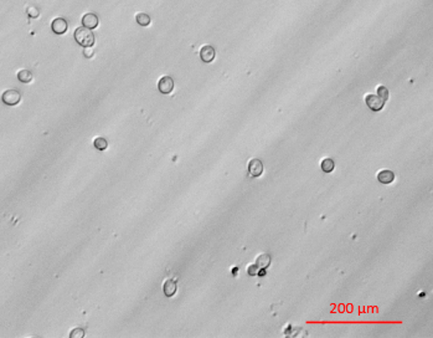 | 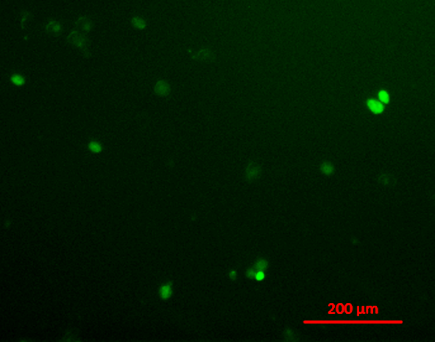 |
| *ScMSN4-P2A-GFP* | 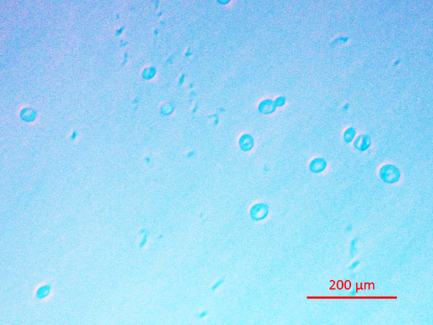 | 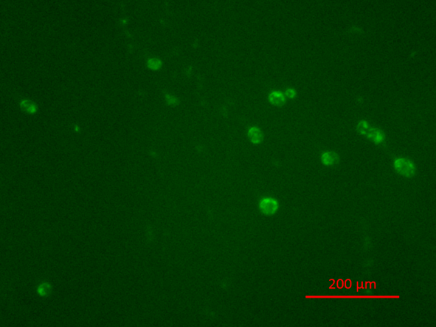 |
| *ScSFP1-P2A-GFP* | 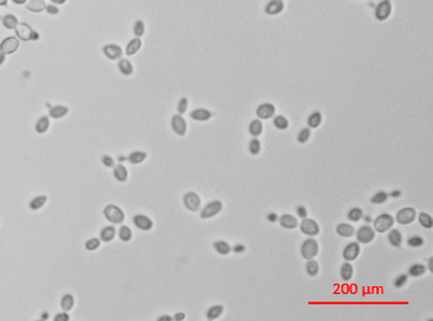 | 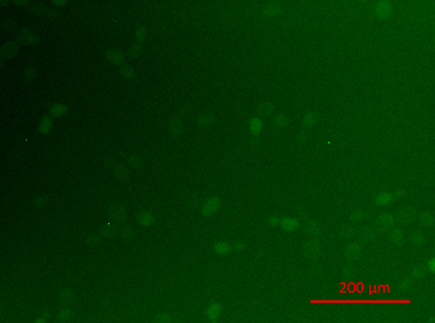 |
| *ScRPN4-P2A-GFP* | 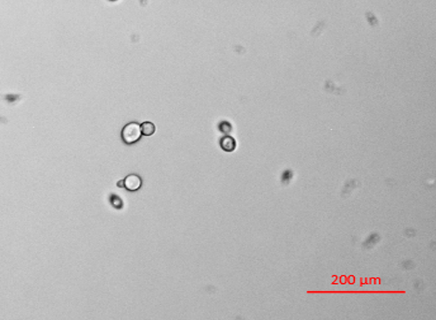 | 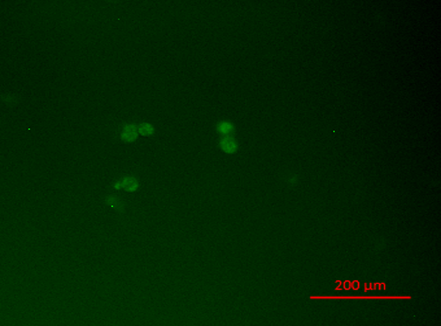 |

| **Expressed gene** | **Bright-field microscopy** | **Fluorescence microscopy** |
| --- | --- | --- |
| *ScGCN4-P2A-GFP* | 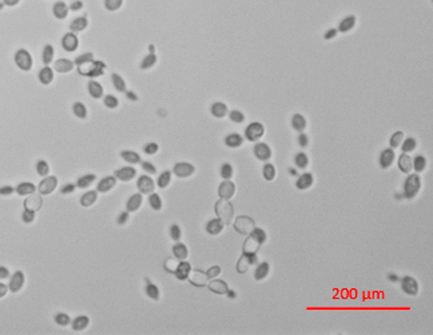 | 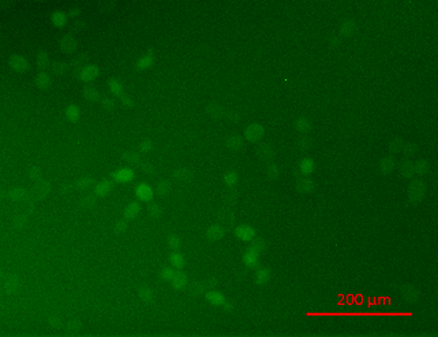 |
| *ScCST6-P2A-GFP* | 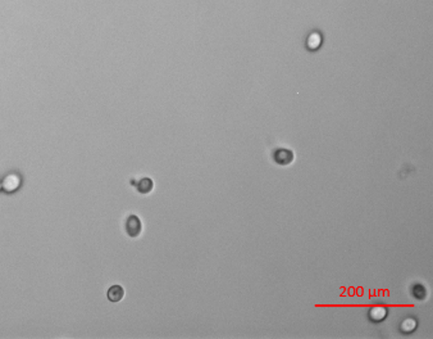 | 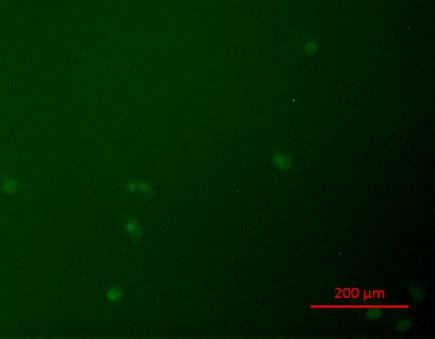 |
| *ScSNF2-P2A-GFP* | 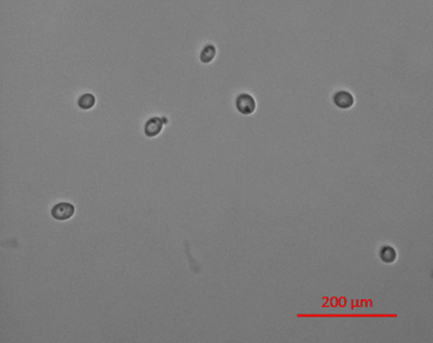 | 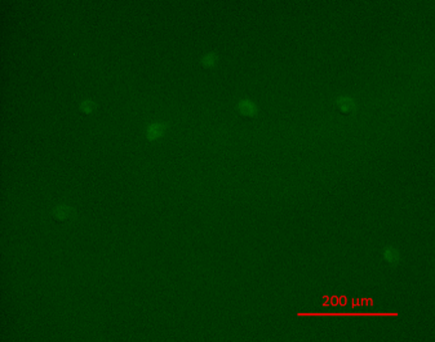 |
| *KmHSF1-P2A-GFP* | 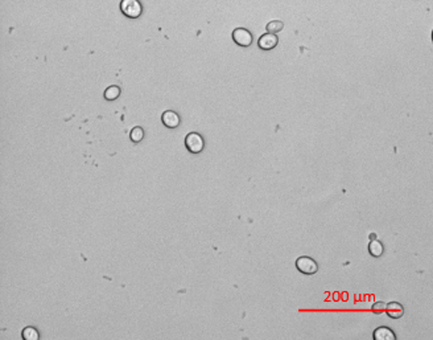 | 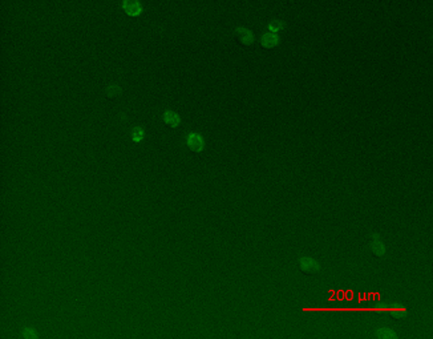 |
| *KmMSN2-P2A-GFP* | 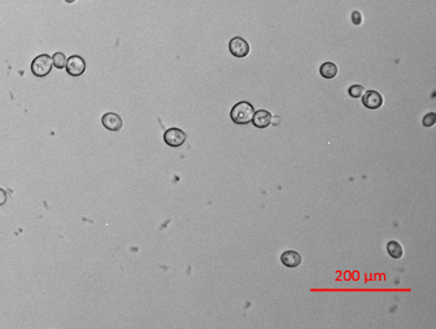 | 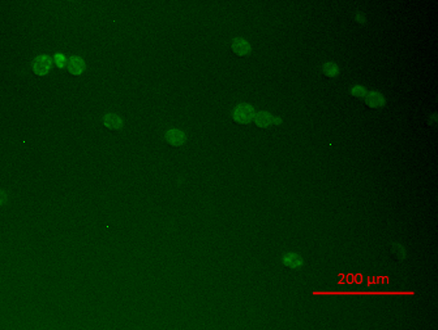 |

| **Expressed gene** | **Bright-field microscopy** | **Fluorescence microscopy** |
| --- | --- | --- |
| *KmSFP1-P2A-GFP* | 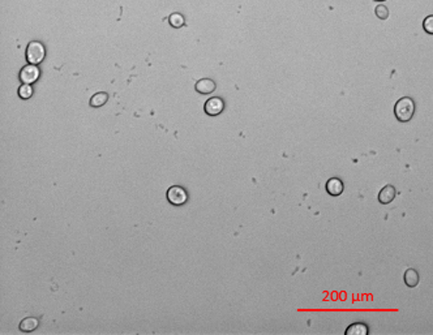 | 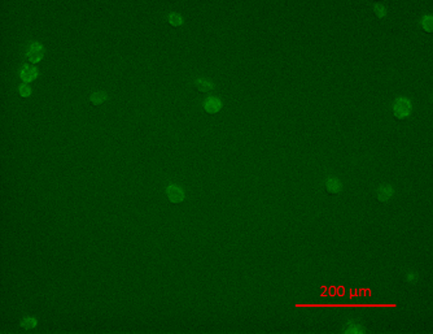 |
| *KmRPN4-P2A-GFP* | 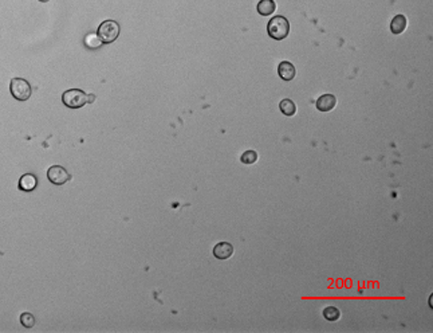 | 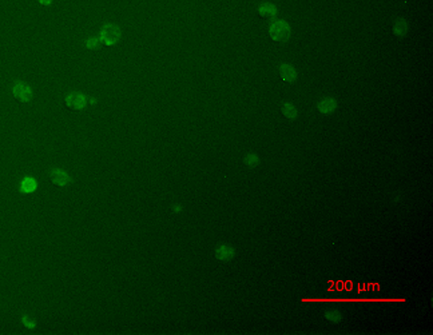 |
| *KmGCN4-P2A-GFP* | 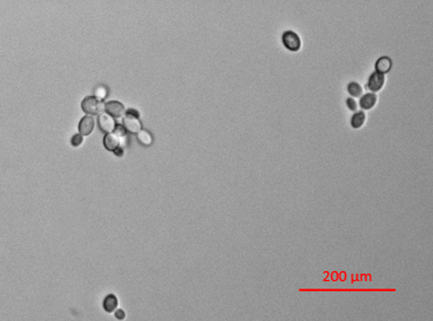 | 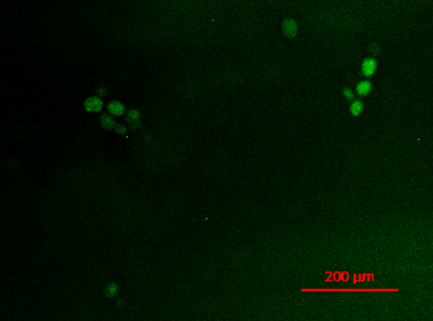 |
| *KmCST6-P2A-GFP* | 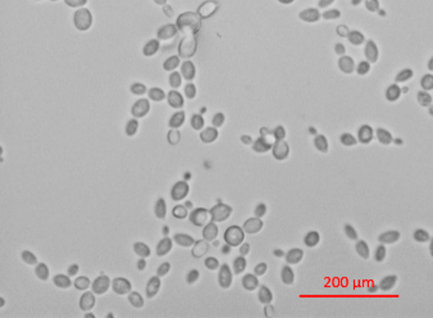 | 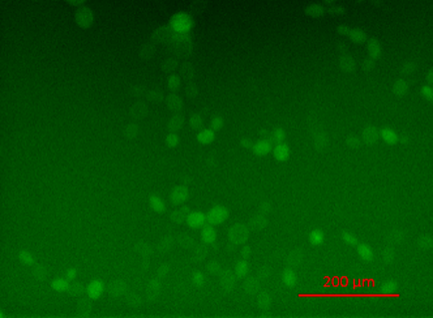 |
| *KmSNF2-P2A-GFP* | 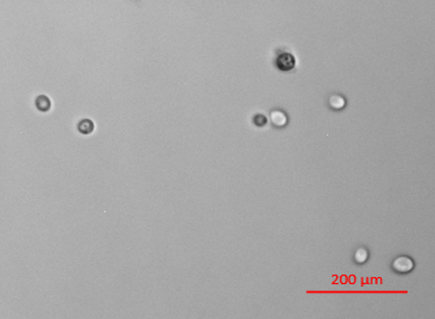 | 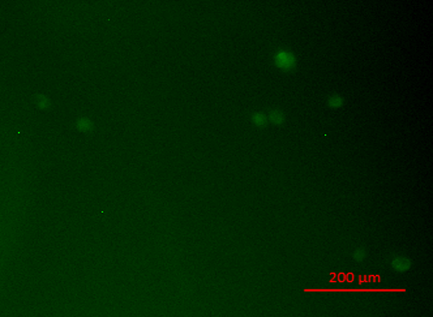 |
