# Supplementary material for: The transcription factors Hsf1 and Msn2 of thermotolerant Kluyveromyces marxianus promote cell growth and ethanol fermentation of Saccharomyces cerevisiae at high temperatures
Source: Biotechnol Biofuels. 2017 Dec 4;10:289. doi: 10.1186/s13068-017-0984-9 (PMC5713069; doi:10.1186/s13068-017-0984-9)
Supplement: Supplementary file 5 — Additional file 5: Table S2. GO analysis of differentially expressed genes for KH_43 vs C_43. Table S3. GO analysis of differentially expressed genes for KM_43 vs C_43. Table S4. KEGG analysis of differentially expressed genes for KM_43 vs C_43. [file 13068_2017_984_MOESM5_ESM.docx]

Table S2 GO analysis of differentially expressed genes for KH_43 vs C_43

| **GO term (GO ID)** | **Gene name** | **Fold change** | **Description** |
| --- | --- | --- | --- |
| Transporter activity (GO:0005215) | *ARR3* | 1.6741514 | Plasma membrane metalloid/H^+^ antiporter; transports arsenite and antimonite; required for resistance to arsenic compounds; transcription is activated by Arr1p in the presence of arsenite [Source:SGD;Acc:S000006405] |
|  | *FCY2* | 1.54801559 | Purine-cytosine permease; mediates purine (adenine, guanine, and hypoxanthine) and cytosine accumulation; relative distribution to the vacuole increases upon DNA replication stress [Source:SGD;Acc:S000000858] |
|  | *OAC1* | 1.53215261 | Mitochondrial inner membrane transporter; transports oxaloacetate, sulfate, thiosulfate, and isopropylmalate; member of the mitochondrial carrier family [Source:SGD;Acc:S000001603] |
|  | *HXT6* | 1.660353 | High-affinity glucose transporter; member of the major facilitator superfamily, nearly identical to Hxt7p, expressed at high basal levels relative to other *HXT*s, repression of expression by high glucose requires *SNF3*; *HXT6* has a paralog, *HXT1*, that arose from the whole genome duplication [Source:SGD;Acc:S000002751] |
|  | *HSP30* | 1.86583318 | Negative regulator of the H(+)-ATPase Pma1p; stress-responsive protein; hydrophobic plasma membrane localized; induced by heat shock, ethanol treatment, weak organic acid, glucose limitation, and entry into stationary phase [Source:SGD;Acc:S000000615] |
|  | *ACB1* | 1.45713041 | Acyl-CoA-binding protein; transports newly synthesized acyl-CoA esters from fatty acid synthetase (Fas1p-Fas2p) to acyl-CoA-consuming processes; subject to starvation-induced, Grh1p-mediated unconventional secretion; protein abundance increases in response to DNA replication stress [Source:SGD;Acc:S000003269] |
|  | *HXT1* | 0.58261308 | Low-affinity glucose transporter of the major facilitator superfamily; expression is induced by Hxk2p in the presence of glucose and repressed by Rgt1p when glucose is limiting; *HXT1* has a paralog, *HXT6*, what arose from the whole genome duplication [Source:SGD;Acc:S000001136] |
|  | *PTR2* | 0.67956808 | Integral membrane peptide transporter; mediates transport of di- and tri-peptides; conserved protein that contains 12 transmembrane domains; PTR2 expression is regulated by the N-end rule pathway via repression by Cup9p [Source:SGD;Acc:S000001801] |
|  | *GNP1* | 0.66416794 | High-affinity glutamine permease; also transports Leu, Ser, Thr, Cys, Met and Asn; expression is fully dependent on Grr1p and modulated by the Ssy1p-Ptr3p-Ssy5p (SPS) sensor of extracellular amino acids; *GNP1* has a paralog, *AGP1*, that arose from the whole genome duplication [Source:SGD;Acc:S000002916] |
|  | *TPO4* | 0.35106255 | Polyamine transporter of the major facilitator superfamily; member of the 12-spanner drug:H(+) antiporter DHA1 family; recognizes spermine, putrescine, and spermidine; localizes to the plasma membrane [Source:SGD;Acc:S000005799] |
|  | *PDR12* | 0.65225138 | Plasma membrane ATP-binding cassette (ABC) transporter; weak-acid-inducible multidrug transporter required for weak organic acid resistance; induced by sorbate and benzoate and regulated by War1p; mutants exhibit sorbate hypersensitivity [Source:SGD;Acc:S000005979] |
|  | *HXT5* | 0.66465151 | Hexose transporter with moderate affinity for glucose; induced in the presence of non-fermentable carbon sources, induced by a decrease in growth rate, contains an extended N-terminal domain relative to other *HXT*s; *HXT5* has a paralog, *HXT3*, that arose from the whole genome duplication [Source:SGD;Acc:S000001138] |
|  | *PDR15* | 0.55221041 | Plasma membrane ATP binding cassette (ABC) transporter; multidrug transporter and general stress response factor implicated in cellular detoxification; regulated by Pdr1p, Pdr3p and Pdr8p; promoter contains a PDR responsive element; *PDR15* has a paralog, PDR5, that arose from the whole genome duplication [Source:SGD;Acc:S000002814] |
|  | *PUT4* | 0.35593853 | Proline permease; required for high-affinity transport of proline; also transports the toxic proline analog azetidine-2-carboxylate (AzC); *PUT4* transcription is repressed in ammonia-grown cells [Source:SGD;Acc:S000005875] |

Table S3 GO analysis of differentially expressed genes for KM_43 vs C_43

| **GO term (GO ID)** | **Gene name** | **Fold change** | **Description** |
| --- | --- | --- | --- |
| Monocarboxylic acid metabolic process (GO:0032787) | *ENO1* | 1.83998 | Enolase I, a phosphopyruvate hydratase; catalyzes conversion of 2-phosphoglycerate to phosphoenolpyruvate during glycolysis and the reverse reaction during gluconeogenesis; expression repressed in response to glucose; protein abundance increases in response to DNA replication stress; N-terminally propionylated in vivo; *ENO1* has a paralog, *ENO2*, that arose from the whole genome duplication [Source:SGD;Acc:S000003486] |
|  | *FAS1* | 1.784401 | Beta subunit of fatty acid synthetase; complex catalyzes the synthesis of long-chain saturated fatty acids; contains acetyl transacylase, dehydratase, enoyl reductase, malonyl transacylase, and palmitoyl transacylase activities [Source:SGD;Acc:S000001665] |
|  | *ADH1* | 1.681501 | Alcohol dehydrogenase; fermentative isozyme active as homo- or heterotetramers; required for the reduction of acetaldehyde to ethanol, the last step in the glycolytic pathway; *ADH1* has a paralog, *ADH5*, that arose from the whole genome duplication [Source:SGD;Acc:S000005446] |
|  | *ERG3* | 1.797759 | C-5 sterol desaturase; glycoprotein that catalyzes the introduction of a C-5(6) double bond into episterol, a precursor in ergosterol biosynthesis; mutants are viable, but cannot grow on non-fermentable carbon sources; substrate of the HRD ubiquitin ligase [Source:SGD;Acc:S000004046] |
|  | *YGP1* | 1.734212 | Cell wall-related secretory glycoprotein; induced by nutrient deprivation-associated growth arrest and upon entry into stationary phase; may be involved in adaptation prior to stationary phase entry; YGP1 has a paralog, SPS100, that arose from the whole genome duplication [Source:SGD;Acc:S000005104] |
|  | *ACB1* | 1.619434 | Acyl-CoA-binding protein; transports newly synthesized acyl-CoA esters from fatty acid synthetase (Fas1p-Fas2p) to acyl-CoA-consuming processes; subject to starvation-induced, Grh1p-mediated unconventional secretion; protein abundance increases in response to DNA replication stress [Source:SGD;Acc:S000003269] |
|  | *PGI1* | 1.545432 | Glycolytic enzyme phosphoglucose isomerase; catalyzes the interconversion of glucose-6-phosphate and fructose-6-phosphate; required for cell cycle progression and completion of the gluconeogenic events of sporulation [Source:SGD;Acc:S000000400] |
|  | *OLE1* | 1.649581 | Delta(9) fatty acid desaturase; required for monounsaturated fatty acid synthesis and for normal distribution of mitochondria [Source:SGD;Acc:S000003023] |
|  | *ALD6* | 0.407649 | Cytosolic aldehyde dehydrogenase; activated by Mg^2+^ and utilizes NADP^+^ as the preferred coenzyme; required for conversion of acetaldehyde to acetate; constitutively expressed; locates to the mitochondrial outer surface upon oxidative stress [Source:SGD;Acc:S000005982] |
| Glucose metabolic process (GO:0006006) | *PGI1* | 1.545432 | Glycolytic enzyme phosphoglucose isomerase; catalyzes the interconversion of glucose-6-phosphate and fructose-6-phosphate; required for cell cycle progression and completion of the gluconeogenic events of sporulation [Source:SGD;Acc:S000000400] |
|  | *ADH1* | 1.681501 | Alcohol dehydrogenase; fermentative isozyme active as homo- or heterotetramers; required for the reduction of acetaldehyde to ethanol, the last step in the glycolytic pathway; *ADH1* has a paralog, *ADH5*, that arose from the whole genome duplication [Source:SGD;Acc:S000005446] |
|  | *TDH3* | 1.912301 | Glyceraldehyde-3-phosphate dehydrogenase (GAPDH), isozyme 3; involved in glycolysis and gluconeogenesis; tetramer that catalyzes the reaction of glyceraldehyde-3-phosphate to 1,3 bis-phosphoglycerate; detected in the cytoplasm and cell wall; GAPDH-derived antimicrobial peptides secreted by *S. cerevisiae* are active against a wide variety of wine-related yeasts and bacteria; binds AU-rich RNA; *TDH3* has a paralog, *TDH2*, that arose from the whole genome duplication [Source:SGD;Acc:S000003424] |
|  | *ENO1* | 1.83998 | Enolase I, a phosphopyruvate hydratase; catalyzes conversion of 2-phosphoglycerate to phosphoenolpyruvate during glycolysis and the reverse reaction during gluconeogenesis; expression repressed in response to glucose; protein abundance increases in response to DNA replication stress; N-terminally propionylated in vivo; *ENO1* has a paralog, *ENO2*, that arose from the whole genome duplication [Source:SGD;Acc:S000003486] |
|  | *TDH1* | 2.218216 | Glyceraldehyde-3-phosphate dehydrogenase (GAPDH), isozyme 1; involved in glycolysis and gluconeogenesis; tetramer that catalyzes the reaction of glyceraldehyde-3-phosphate to 1,3 bis-phosphoglycerate; detected in the cytoplasm and cell wall; protein abundance increases in response to DNA replication stress; GAPDH-derived antimicrobial peptides secreted by *S. cerevisiae* are active against a wide variety of wine-related yeasts and bacteria [Source:SGD;Acc:S000003588] |
| Monocarboxylic acid biosynthetic process (GO:0072330) | *ACC1* | 1.648187 | Acetyl-CoA carboxylase, biotin containing enzyme; catalyzes carboxylation of cytosolic acetyl-CoA to form malonyl-CoA and regulates histone acetylation by regulating the availability of acetyl-CoA; required for de novo biosynthesis of long-chain fatty acids; ACC1 has a paralog, HFA1, that arose from the whole genome duplication [Source:SGD;Acc:S000005299] |
|  | *ERG3* | 1.797759 | C-5 sterol desaturase; glycoprotein that catalyzes the introduction of a C-5(6) double bond into episterol, a precursor in ergosterol biosynthesis; mutants are viable, but cannot grow on non-fermentable carbon sources; substrate of the HRD ubiquitin ligase [Source:SGD;Acc:S000004046] |
|  | *FAS1* | 1.784401 | Beta subunit of fatty acid synthetase; complex catalyzes the synthesis of long-chain saturated fatty acids; contains acetyltransacylase, dehydratase, enoyl reductase, malonyl transacylase, and palmitoyl transacylase activities [Source:SGD;Acc:S000001665] |
|  | *OLE1* | 1.649581 | Delta(9) fatty acid desaturase; required for monounsaturated fatty acid synthesis and for normal distribution of mitochondria [Source:SGD;Acc:S000003023] |
|  | *ALD6* | 0.407649 | Cytosolic aldehyde dehydrogenase; activated by Mg^2+^ and utilizes NADP^+^ as the preferred coenzyme; required for conversion of acetaldehyde to acetate; constitutively expressed; locates to the mitochondrial outer surface upon oxidative stress [Source:SGD;Acc:S000005982] |

Table S4 KEGG analysis of differentially expressed genes for KM_43 vs C_43

| **KEGG pathway** | **Gene name** | **Fold change** | **Description** |
| --- | --- | --- | --- |
| Glycolysis / Gluconeogenesis (sce00010) | *ENO1* | 1.839979892 | Enolase I, a phosphopyruvate hydratase; catalyzes conversion of 2-phosphoglycerate to phosphoenolpyruvate during glycolysis and the reverse reaction during gluconeogenesis; expression repressed in response to glucose; protein abundance increases in response to DNA replication stress; N-terminally propionylated in vivo; *ENO1* has a paralog, *ENO2*, that arose from the whole genome duplication [Source:SGD;Acc:S000003486] |
|  | *TDH1* | 2.218216222 | Glyceraldehyde-3-phosphate dehydrogenase (GAPDH), isozyme 1; involved in glycolysis and gluconeogenesis; tetramer that catalyzes the reaction of glyceraldehyde-3-phosphate to 1,3 bis-phosphoglycerate; detected in the cytoplasm and cell wall; protein abundance increases in response to DNA replication stress; GAPDH-derived antimicrobial peptides secreted by *S. cerevisiae* are active against a wide variety of wine-related yeasts and bacteria [Source:SGD;Acc:S000003588] |
|  | *PGI1* | 1.545431811 | Glycolytic enzyme phosphoglucose isomerase; catalyzes the interconversion of glucose-6-phosphate and fructose-6-phosphate; required for cell cycle progression and completion of the gluconeogenic events of sporulation [Source:SGD;Acc:S000000400] |
|  | *ADH1* | 1.681501423 | Alcohol dehydrogenase; fermentative isozyme active as homo- or heterotetramers; required for the reduction of acetaldehyde to ethanol, the last step in the glycolytic pathway; *ADH1* has a paralog, *ADH5*, that arose from the whole genome duplication [Source:SGD;Acc:S000005446] |
|  | *TDH3* | 1.912301498 | Glyceraldehyde-3-phosphate dehydrogenase (GAPDH), isozyme 3; involved in glycolysis and gluconeogenesis; tetramer that catalyzes the reaction of glyceraldehyde-3-phosphate to 1,3 bis-phosphoglycerate; detected in the cytoplasm and cell wall; GAPDH-derived antimicrobial peptides secreted by *S. cerevisiae* are active against a wide variety of wine-related yeasts and bacteria; binds AU-rich RNA; *TDH3* has a paralog, *TDH2*, that arose from the whole genome duplication [Source:SGD;Acc:S000003424] |
|  | *PDC6* | 0.407479674 | Minor isoform of pyruvate decarboxylase; decarboxylates pyruvate to acetaldehyde, involved in amino acid catabolism; transcription is glucose- and ethanol-dependent, and is strongly induced during sulfur limitation [Source:SGD;Acc:S000003319] |
|  | *TPI1* | 1.640288973 | Triose phosphate isomerase, abundant glycolytic enzyme; mRNA half-life is regulated by iron availability; transcription is controlled by activators Reb1p, Gcr1p, and Rap1p through binding sites in the 5' non-coding region; inhibition of Tpi1p activity by PEP (phosphoenolpyruvate) stimulates redox metabolism in respiring cells; E104D mutation in human TPI causes a rare autosomal disease [Source:SGD;Acc:S000002457] |
|  | *ALD6* | 0.407649175 | Cytosolic aldehyde dehydrogenase; activated by Mg^2+^ and utilizes NADP^+^ as the preferred coenzyme; required for conversion of acetaldehyde to acetate; constitutively expressed; locates to the mitochondrial outer surface upon oxidative stress [Source:SGD;Acc:S000005982] |
